# Development of a composite drought indicator for operational drought monitoring in the MENA region

**DOI:** 10.1038/s41598-024-55626-0

**Published:** 2024-03-05

**Authors:** Karim Bergaoui, Makram Belhaj Fraj, Stephen Fragaszy, Ali Ghanim, Omar Hamadin, Emad Al-Karablieh, Jawad Al-Bakri, Mona Fakih, Abbas Fayad, Fadi Comair, Mohamed Yessef, Hayat Ben Mansour, Haythem Belgrissi, Kristi Arsenault, Christa Peters-Lidard, Sujay Kumar, Abheera Hazra, Wanshu Nie, Michael Hayes, Mark Svoboda, Rachael McDonnell

**Affiliations:** 1 International Water Management Institute (IWMI), Colombo, Sri Lanka; 2 Dubai Technology Entrepreneur Campus, ACQUATEC Solutions, Dubai, UAE; 3 Drought Management Unit, Ministry of Water and Irrigation, Amman, Jordan; 4 Jordanian Meteorological Department, Ministry of Transportation, Amman, Jordan; 5 Department of Agricultural Economics and Agribusiness, The University of Jordan, Amman, Jordan; 6 Department of Land, Water and Environment, The University of Jordan, Amman, Jordan; 7 Water Resources, General Directorate of Hydraulic and Electrical Resources, Ministry of Energy and Water, Beirut, Lebanon; 8 Centre for Hydrology, University of Saskatchewan, Canmore, Alberta T1W 3G1 Canada; 9 Energy, Environment, and Water Research Centre in the Cyprus Institute, Nicosia, Cyprus; 10 Institut Hassan II of Agronomy and Veterinary Medicine, Rabat, Morocco; 11 Direction Générale des Ressources en Eau, Tunis, Tunisia; 12 Institut National de la Météorologie, Tunis, Tunisia; 13 Hydrological Science Laboratory, NASA Goddard Space Flight Center, Greenbelt, MD USA; 14 Earth System Science Interdisciplinary Center, University of Maryland, Maryland, USA; 15 NASA Goddard Space Flight Center, Maryland, USA; 16 Earth Science Division, NASA Goddard Space Flight Center, Greenbelt, MD USA; 17 Science Applications International Corporation, McLean, VA USA; 18 School of Natural Resources, University of Nebraska–Lincoln, Lincoln, NE USA; 19 National Drought Mitigation Center, University of Nebraska–Lincoln, Lincoln, NE USA

**Keywords:** Environmental sciences, Environmental social sciences, Natural hazards

## Abstract

This paper presents the composite drought indicator (CDI) that Jordanian, Lebanese, Moroccan, and Tunisian government agencies now produce monthly to support operational drought management decision making, and it describes their iterative co-development processes. The CDI is primarily intended to monitor agricultural and ecological drought on a seasonal time scale. It uses remote sensing and modelled data inputs, and it reflects anomalies in precipitation, vegetation, soil moisture, and evapotranspiration. Following quantitative and qualitative validation assessments, engagements with policymakers, and consideration of agencies’ technical and institutional capabilities and constraints, we made changes to CDI input data, modelling procedures, and integration to tailor the system for each national context. We summarize validation results, drought modelling challenges and how we overcame them through CDI improvements, and we describe the monthly CDI production process and outputs. Finally, we synthesize procedural and technical aspects of CDI development and reflect on the constraints we faced as well as trade-offs made to optimize the CDI for operational monitoring to support policy decision-making—including aspects of salience, credibility, and legitimacy—within each national context.

## Introduction

The operational identification and quantification of drought location, onset, severity, duration, and recovery is a complex problem due to both scientific and normative challenges. Resource allocation for drought management is politically contentious, and governmental decision-making on what type(s) of drought to monitor regularly and how, and manage proactively, is a values-driven and political choice that empirical evidence can inform^1,2^. Governmental and non-governmental stakeholders in Middle East and North Africa (MENA) countries consider agricultural drought to be the first priority for improved national capacity in drought risk management, with hydrological drought a close second^3,4^.

Most MENA governments monitor drought operationally using the Standardized Precipitation Index (SPI-3) calculated from agro-meteorological observation sites^5^. However, challenges arise due to the distribution and density of stations, as well as issues with data accessibility, record, and quality control^3^.

MENA governments acknowledge these issues, and at the WMO-convened High-Level Meeting on National Drought Policy in 2013, they requested technical support to improve drought risk management systems. Through the resultant USAID-funded MENAdrought project [https://menadrought.iwmi.org/], applied researchers developed a common general modelling framework for drought monitoring and then tested that system and tailored it to meet the requirements of government agencies in four countries (Jordan, Lebanon, Morocco, and Tunisia).

This paper presents the Composite Drought Indicator (CDI) that is now produced monthly by those governments and integrated into Drought Action Plans used by national or basin agencies^6^. The CDI reflects anomalies in precipitation, vegetation, soil moisture, and evapotranspiration using a convergence of evidence approach^7,8^ that is becoming increasingly common in national and regional drought monitoring systems the world^9,10^. It has already been used by agencies to support drought management responses and resource allocation; for example, in early 2022, information from the CDI was used to target, size, and justify the provision of drought relief nationally in Morocco^11^ and in the Tafilah Governorate of Jordan^12^.

The paper also describes the iterative validation and co-development process that:

- shaped the technical system’s refinements overall and for each country to reflect agencies’ constraints (modelling and institutional) and needs (technical and policy), and
- enabled officials—and the agencies they represent—to become confident enough in the resultant system to use it for operational drought management decision support.

As such, the paper focuses on the scientific and technical end of the science-policy interface. It describes chronological development and procedural aspects to highlight the multi-faceted and interactive requirements for development of applied and policy-relevant environmental monitoring tools, particularly in the Global South. Case studies demonstrating how to go about developing and embedding such tools for climate extreme management in operational contexts are critical to support climate change adaptation and achieve the goals of the United Nations Framework Convention on Climate Change (UNFCCC) and Sendai Framework^13^. Considerations, approaches, methods, processes, and/or findings should be transferable to socio-economically, governmentally, and/or agro-ecologically comparable countries, if not more universally.

### Defining needs—CDI requirements and the science–policy interface

In each country, government agencies and other stakeholders described a range of requirements for drought monitoring generally^3^, and the CDI specifically^6,14^:

- First priority is monitoring agricultural drought and particularly rainfed systems for cereals, rangelands, olives, and legumes;
- Output temporal requirements—monthly or more frequently;
- Output spatial requirements—adequate spatial resolution to capture major agricultural and hydrological basins and shifts in agro-ecological zones;
- Simplicity and ease of production and use—must be producible monthly by national agencies, taking into account computing and modelling requirements, staff capacity, and internet bandwidth; and
- Adequate accuracy and precision for drought management policy decision-making.

These needs shaped the CDI development process. They also shaped stakeholders’ considerations of the CDI’s credibility (accuracy), salience (usefulness for decision-making), legitimacy (alignment with users’ values and views), and accessibility, characteristics that are critical for stakeholders’ application of scientific information in policy processes^2,15,16^. Throughout this paper, we identify in italics which of these characteristics factored into specific decision-making for CDI assessment and/or development.

### Iterative co-development—CDI development stages and role of CDI validation

**Figure 1:**
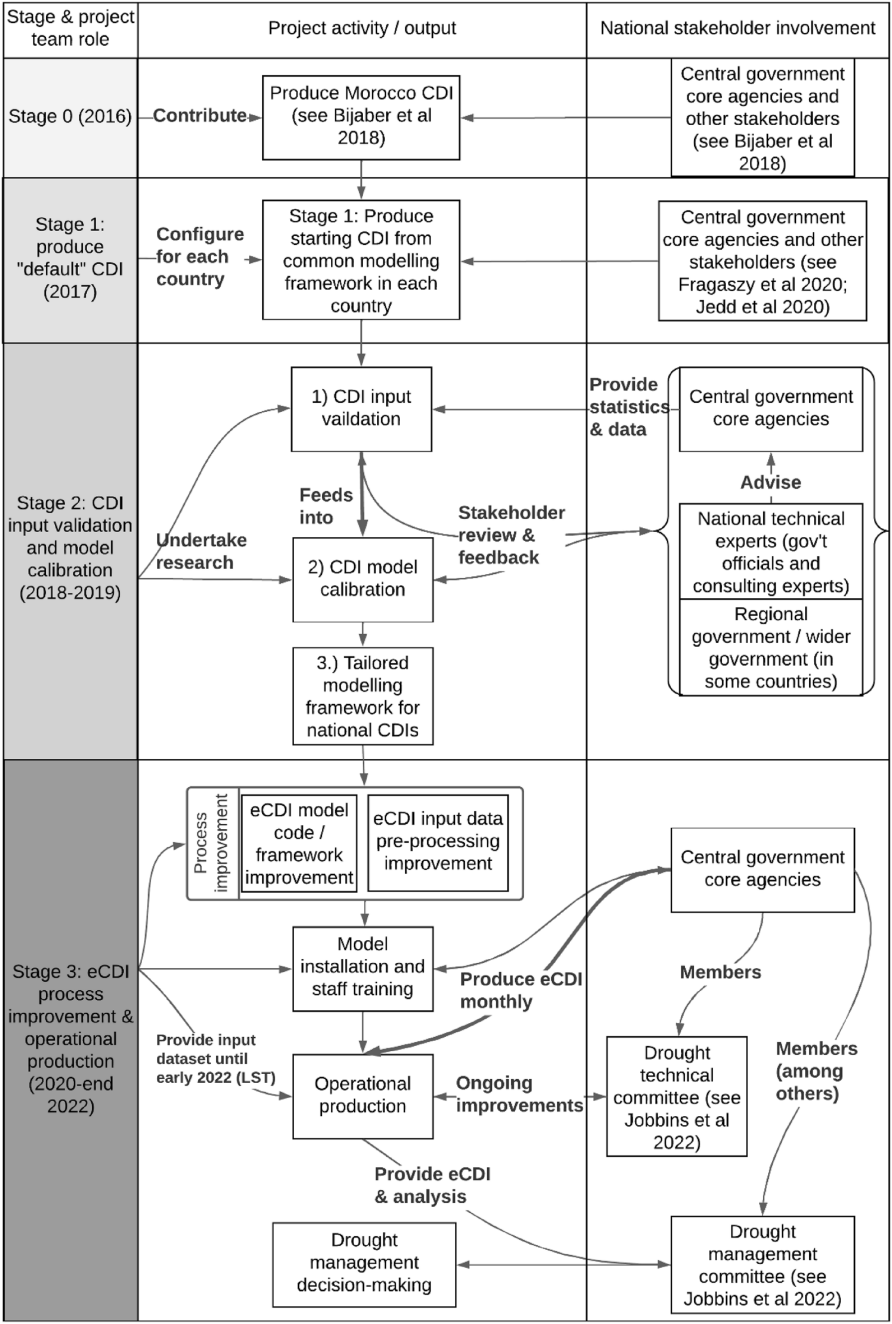
Stages in operational CDI development.

National agencies were closely involved and/or led CDI co-development workstreams in each Stage, as shown in Fig. 1*(legitimacy*). Stage 0 predates the MENAdrought project and resulted in a CDI for Morocco^17^. In Stage 1, we replicated that system in other countries, assessed stakeholder needs, and tested CDI outputs in national workshops^3,4^.

This paper focuses primarily on Stages 2 and 3, and unless otherwise specified, mentions of ‘the CDI’ refer to the version now produced by agencies.

In Stage 2, we undertook a range of validation studies and in Stage 3 iterative refinements to the CDI modelling and production process based on those findings as well as stakeholders’ needs and constraints. Rykiel^18^ defines model validation as “demonstration that a model within its domain of applicability possesses a satisfactory range of accuracy consistent with the intended application of the model”. Validation therefore has empirical/objective and normative/subjective components related to the criteria noted above. In particular, the “range of accuracy” aspect is primarily objective and related to the information’s *credibility* and *salience*; whereas the “satisfactory” and “intended application” aspects are normative and related to *salience* and *legitimacy*.

The process and forms of validation we undertook reflect this, which opened the evaluative space for integration of multiple usable knowledge types^19^. The validation efforts aimed to determine, and subsequently improve, the CDI’s performance in relation to the accuracy of the information it provides (reduce error and make it more *credible*) as well as the utility of the information for guiding drought-related action (increase *salience* and *legitimacy*;^15^) according to stakeholder requirements.

Following Stage 3, national agencies now produce the CDI monthly and provide it to inter-agency technical and management committees (*salience*,^6^). In Morocco they are also made public [https://www.google.com/maps/d/viewer?mid=1z0hdz3xuU8e0ogvev3grFsC_jhwHc85L&ll=31.654976915646525%2C-2.4285752472437583&z=5], albeit with a delay.

## Methods and data for CDI production

Requirements noted in Section "Defining needs—CDI requirements and the science-policy interface" constrained our possible choices for CDI development. Review of in-situ monitoring data availability, quality, coverage, and accessibility in each country (partially reported in^3^) indicated that it would not be possible to produce a CDI monthly with adequate national coverage using that observational data in any of the countries. Paucity of data beyond precipitation, temporal coverage of data, and timeliness of precipitation data collection and collation were the most immediate barriers.

As a result, we opted to rely entirely on an ensemble of satellite remote sensing and modelled data for operational CDI production (*accessibility*).

In this section, we summarily describe:

1. CDI input indices and weighting;
2. CDI input index calculation;
3. CDI integration and ranking using sliding windows; and
4. CDI dissemination using a web interface.

Note that Section "CDI validation findings and iterative refinements to the CDI" also includes summary descriptions of validation methods.

### CDI input indices and weighting

CDI input indices and weightings were initially based on the CDI developed by Bijaber et al. (2018) due to its agricultural drought focus. They were subsequently validated and refined with stakeholders (see Section "CDI validation findings and iterative refinements to the CDI";^3,4,14^) and by the conclusion of Stage 1 had settled into their current form:

- 3 months Standardized Precipitation Index (SPI-3; 40%;^20^);
- Normalized Difference Vegetation Index anomaly (NDVI; 20%;^21^);
- Root zone soil moisture anomaly (hereafter, SMA; 20%;^22^); and
- Day-night land surface temperature amplitude anomaly (hereafter, diurnal LST; 20%;^23^).

The first three indices are widely used for agricultural drought monitoring (*salience, legitimacy*;^24^), and the fourth is an effective proxy for evapotranspiration (relative error of 5 to 10% according to^23^), which is also commonly used. In our context, diurnal LST was substantially easier to calculate, and potentially more accurate, than calculating evapotranspiration anomalies directly (*accessibility*).

### CDI input index calculation

The methods and data for calculation of CDI input indices are primarily shown in Table 1. It includes the specific calculation method for each input index as well as its data source, latency, period of record, temporal and spatial scale, and any additional data filtering or processing. Section "Iterative development of precipitation inputs" provides rationale for the difference in SPI-3 production across the countries.

**Table 1:** –CDI input index calculation. Asterisks (*) denote the system as it is prepared for implementation following operational monthly availability of the relevant VIIRS data.

Index	Data inputs	Additional filtering or processing	Data latency	Temporal scale of data	Spatial scale of data	Index calculation for use in CDI (baseline period for all is 2000–2020)
SPI-3^20^	Jordan: IMERG^25,26^ (See NASA GES DISC: https://gpm1.gesdisc.eosdis.nasa.gov/data/GPM_L3/GPM_3IMERGDL.06/)	None	1–2 days after end of month	Daily	10 km × 10 km; resampled to 5 km × 5 km using a simple bilinear interpolation method^27^	*Step 1*: Produce daily values *Step 2*: Sum daily values across each month as represented by 6 different values deduced from a sliding window of two days difference starting from the beginning of the month *Step 3*: Calculate SPI-3 *Step 4*: Calculate SPI-3 percentiles per month across whole period (2000–2020)
	Lebanon and Tunisia: CHIRPS final^28^ (CHIRPS preliminary and final product: https://data.chc.ucsb.edu/products/CHIRPS-2.0/global_daily/tifs/p05/)	None	2–3 weeks after end of month	Daily	5 km × 5 km	Same as above
	Morocco : CHIRPS preliminary (Ibid)	Production of ‘estimated CHIRPS final product’ using CNN models (see Supplementary Information A)	1–2 days after end of month	Daily	5 km × 5 km	Same as for Jordan following the addition of a new Step 1: Provide CHIRPS preliminary data and IMERG predictors data to CNN model to produce estimated CHIRPS final data
NDVI	Current system: eMODIS, download every 5 days from earth explorer: (https://earthexplorer.usgs.gov/ EROS Moderate Resolution Imaging Spectroradiometer (eMODIS) Digital Object Identifier (DOI) number: /10.5066/F7H41PNT) Prepared system*: VIIRS download every 5 days (VIIRS NDVI: https://e4ftl01.cr.usgs.gov/VIIRS/)	Use cloud mask & quality control filters on eMODIS data; additional cloud masking and daily interpolation using Savitzky-Golay filters (see 2.2.3)	5 days	Average of 10 days	250 m × 250 m (current system) and 375 m × 375 m (prepared system); both aggregated at 5 km × 5 km	*Step 1*: we apply a 93-days window and a polynomial degree p = 1 on each 250-m pixel *Step 2*: Interpolate daily NDVI data from Savitsky-Golay filter^29^ *Step 3*: Produce monthly average NDVI values, and percentiles of these, as per Steps 2 and 4 shown for SPI-3
Diurnal LST	Current system: MODIS/ Terra (MOD11A1 Collection 6) daily download ( MODIS/ Terra (MOD11A1 Collection 6): https://e4ftl01.cr.usgs.gov/MOLT/MOD11C1.006/); LIS-Noah-MP (Ibid.) Prepared system: VIIRS daily download*	Use of cloud mask & quality control filters on MODIS data; additional cloud masking and model gap-filling (see 2.2.2)	4 h	Daily	1 km × 1 km (current system), and 375 m × 375 (prepared system); both aggregated at 5 km × 5 km	*Step 1*: Collect and produce modelled daily values of day-night temperature amplitude (see 2.2.2) *Step 2*: Produce monthly average day-night temperature amplitude data, and percentiles of these, as per Steps 2 and 4 shown for SPI-3
SMA	LIS-Noah-MP V7.2^30,31^, GDAS forcings download every 6 h from NASA portal: (https://portal.nccs.nasa.gov/lisdata_pub/data/MET_FORCING/GDAS/)	dynamic phenology module^32^	Model output	15-min time-steps	1 km × 1 km; aggregated to 5 km × 5 km	Absolute values of root zone soil moisture (from 4 layers) are a model output *Step 1*: Produce daily soil moisture data by averaging soil moisture across all layers *Step 2*: Produce monthly average soil moisture values, and percentiles of these, as per Steps 2 and 4 shown for SPI-3

In the remainder of this sub-section, we describe:

- Noah-MP model establishment and parameterization (used for SMA and diurnal LST);
- A novel application of harmonic analysis and Fourier transformations for cloud masking and data fusion for gap-filling related to diurnal LST; and
- Cloud masking, gap-filling, and data smoothing using Savitzky-Golay filters for NDVI.

Supplementary Information A provides additional details on (1) the novel harmonic analysis and Fourier transformation method and results, (2) development and use of convolutional neural network models for SPI-3 production in Morocco, and (3) NDVI gap-filling results.

#### Establishment and parameterization of the Noah-MP modelling framework

The SMA and diurnal LST indices are based on outputs of the LIS modelling framework^30^ running the multiple parameterizations land surface model Noah-MP^31^.

We used the dynamic phenology model within Noah-MP to account for plant processes that respond to cold, heat, and drought stresses^32^. The model simulates leaf area index due to senescence or herbivory. Therefore, during drought periods, the green vegetation fraction lowers and produces feedbacks to increase surface temperature and further reduce soil moisture. This was the major refinement made to SMA production in Stage 3.

We used input parameters including the MODIS-IGBP land cover data^33^, STATSGO & FAO soil texture data^34,35^, and SRTM for elevation and slope ^36^. The model was forced with Global Data Assimilation System (GDAS) data [National Climatic Data Center, NESDIS, NOAA, U.S. Department of Commerce Dataset Title: NCEP EDAS and GDAS (FNL) Model Data (DSI-6141); [^37^] for temperature, relative humidity, wind, rainfall and downward surface solar radiation. The model was spun up using 4 cycles per day (at 0000. 0600. 1200. and 1800 UTC) covering the period between 2000 and 2015.

#### Novel application of harmonic analysis and Fourier transformations for cloud masking and gap-filling with data fusion

MODIS LST data have issues with cloud masking even after the application of quality coefficients^38^, especially cloud edges and thin clouds, which are the major sources of contamination^39^. Figure A4 exemplifies the extensiveness of this problem.

To overcome these challenges, we applied a new method that combines remotely sensed data and model gap-filling: we used harmonic analysis and Fourier transformations to identify cloud-affected pixels and gap-fill temperature data reconstructed from quality-controlled MODIS values and Noah-MP model outputs.

Firstly, we used the Harmonic ANalysis of Time Series (HANTS) algorithm^40–42^ on MODIS land surface temperature values (T_s_) to produce a new, smoothed time series (T_s_^hants^). T_s_ values that deviated more than 4 °C from the T_s_^hants^ were flagged as cloud affected and removed from the clean dataset. We decided on the 4 °C threshold after visually examining cloudy scenes from multiple regions and times from 2015 data, and then using expert judgment alone due to data scarcity for model validation.

Then cloud affected pixels were replaced by updated estimates of T_s_ as follows:

*Step 1:* create a “daily perturbation” model (dT_obs_) for MODIS-observed T_s_ and smoothed T_s_^hants^ such that dT_obs_ = T_s_ – T_s_^hants^.

*Step 2:* simulate LST values (T_s model_) using Noah-MP and produce a smoothed time series (T_s_^hants model^). We then produce a daily perturbation model (dT_model_) of the simulated values and smoothed time series as shown in Step 1.

*Step 3:* establish a relationship between dT_obs_ and dT_model_ through a simple linear regression analysis.

*Step 4:* estimate dT_obs_ for clear and cloudy days as follows:

1 $$\text{dT}_{\text{obs}} = \text{a} * \text{dT}_{\text{model}} + \text{b}$$

In Eq. 1, a and b are the regression coefficients between dT_modis_ and dT_obs_.

*Step 5:* For cloud-affected pixels, there is no value of T_s_ except the harmonic estimate of T_s_^hants^. By producing daily values of dT_obs_ from Eq. 1, we can realistically correct the smoothed T_s_^hants^ and reconstruct temperature data (T_s_^reconst^) for the missing cloudy days as follows:

2 $$\text{T}_{\text{s}}^{\text{reconst}} = \text{T}_{\text{s}}^{\text{hants}} + a * \text{dT}_{\text{obs}} + \text{b}$$

*Step 6:* Repeat all steps each month for CDI production because regression coefficients change.

The presented gap-filling method does not rely on the absolute values of modelled temperature. It thus avoids known modelling errors in quantifying absolute values and smoothed time series of LST. Rather, this method relies on the model to simulate daily variation of LST. More detail and pseudocode are shown in Supplementary Information A.

#### Improving NDVI outputs using Savitzky–Golay filters

Like for LST, eMODIS and VIIRS NDVI products have issues with cloud masking even after the application of quality coefficients [^38,39^]. For the Stage 1 CDI, despite only using quality-controlled eMODIS data, there were numerous and extensive instances of cloud cover impacts. These materialize as sudden, very low NDVI values in the quality-controlled time series. Also, the eMODIS suite of products includes 10-day composite datasets that are updated every five days.

To reconstruct a better NDVI time-series dataset without missing pixel values (cloud-affected), and to reduce noise, we used Savitzky-Golay filters to produce cloud free and daily interpolated NDVI time-series^29,43^. The filtered values have no cloud effect while still conserving the seasonal NDVI minima and maxima. Figure A5 shows an example of the effect from a pixel in Tall Zanoub, southern Lebanon.

### CDI integration and ranking using sliding windows

To calculate the CDI, we first normalize the four components through percentile ranking. We used a sliding window approach^44^ to produce extra statistical datapoints with the objective of providing more realistic ranking results (*credibility, salience*) by smoothing fluctuations and helping identify extreme events (e.g.,^45^).

The sliding window approach generates six representations of the monthly value for each input index (and the integrated CDI). CDI_6_ represents the full month (e.g., 1–31 May), CDI_5_ is offset two days earlier (ie., 29 April–29 May), etc., with CDI_1_ being the full month offset twelve days earlier. This results in more than 120 total values for each month over the climatological baseline period (six values per month per year since February 2000, that date from which requisite satellite data is available).

We then use a linear model for CDI index integration as shown in Eq. 1 because it is easy to understand, simple to calculate, and has proven effective elsewhere (e.g.,^46^; *legitimacy, accessibility*).

3 $$\text{CDI}_{\text{i}\left( {1 - 6} \right)} = \text{a}_{1 \,(40\% )}\text{SPI-3}_{\text{i}} + \text{a}_{2\,(20\%)}\text{NDVI}_{\text{i}} + \text{a}_{3\,(20\% )}\text{SMA}_{\text{i}} + \text{a}_{4\, (20\% )}\,\text{diurnal LST}_{\text{i}}$$

Finally, each month, we rank solely CDI_6_ with the ranking based on values of CDI_1-6_ of that month of the whole period. We roughly follow drought class definitions of the U.S. Drought Monitor^7^:

- D0—“normal” with CDI over 20th percentile;
- D1—“moderate drought” with CDI between 10 and 20th percentiles;
- D2—“severe drought” with CDI between 2nd and 10th percentiles;
- D3—“extreme drought” with CDI under 2nd percentile.

**Figure 2:**
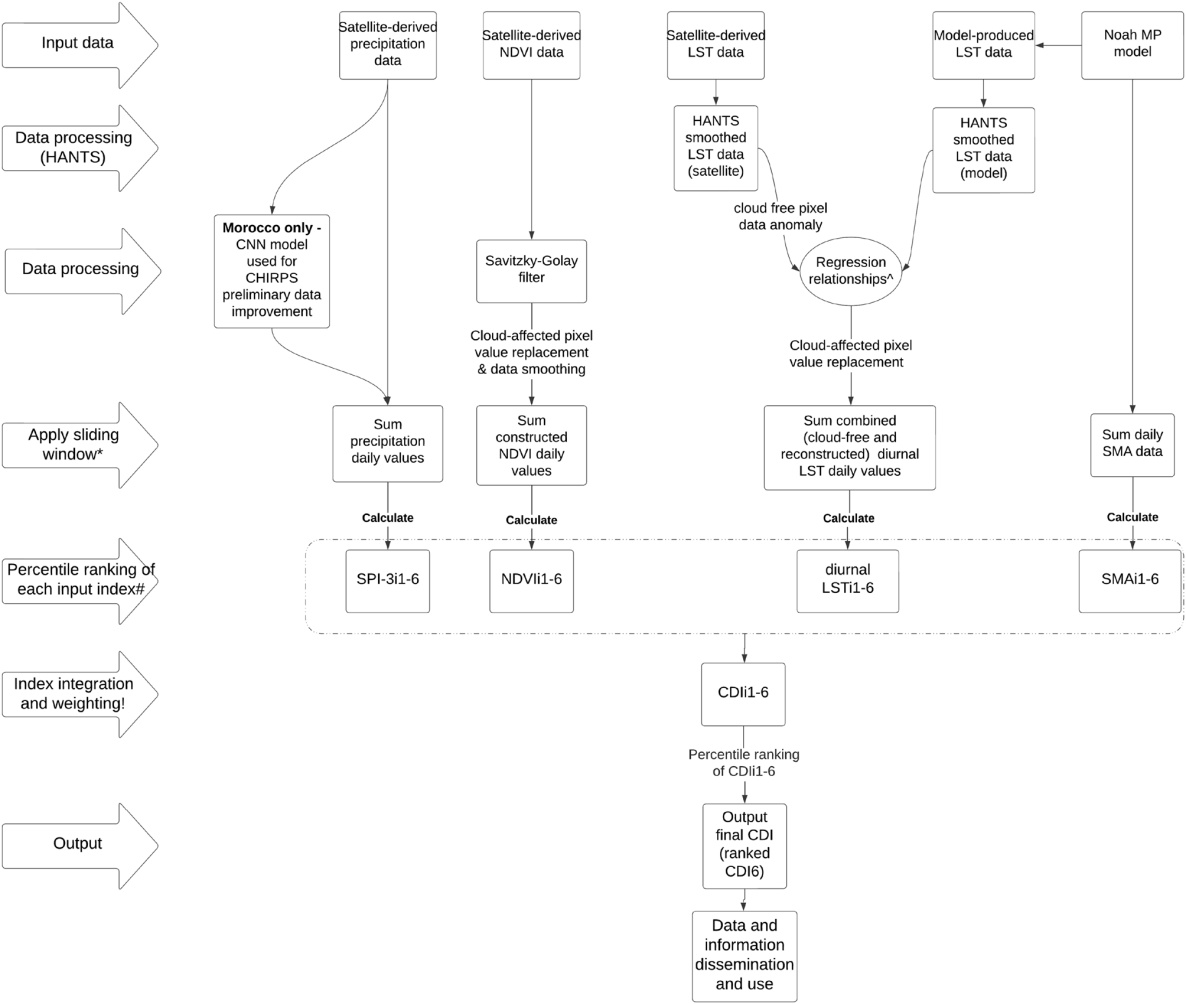
Conceptual visualization of input index production and CDI integration. ^^^Regression relationships of (1) difference between observed (satellite) and HANTS-smoothed (satellite) pixel data for the entire year, and (2) difference between LST model and HANTS-smoothed (LST model) pixel data for the entire year. *Sliding windows applied to daily values of data for each input index. ^#^Percentile ranking of each input index based on climatological record from all monthly sliding window outputs (ie., SPI-3_i1-6_ for each month from 2000 to 2022). ^!^Percentile ranking of integrated CDI values based on all from every integrated CDI sliding window output (ie., CDI_i1-6_ for each month from 2000 to 2022).

**Figure 3:**
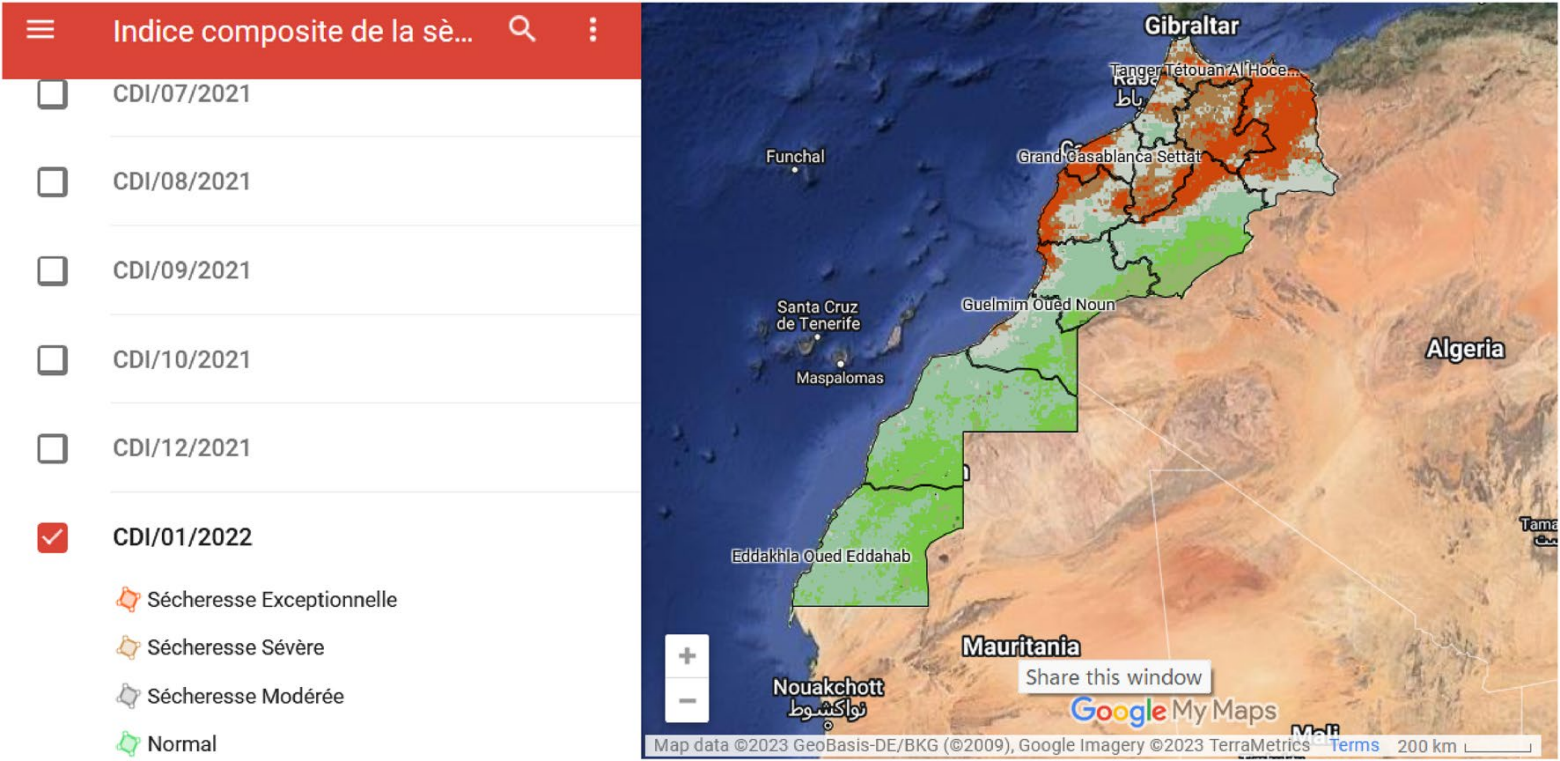
Screenshot of Moroccan CDI outputs for January 2022 published by the DSS. Source: https://www.google.com/maps/d/u/0/viewer?mid=1z0hdz3xuU8e0ogvev3grFsC_jhwHc85L&ll=27.58758794976649%2C-5.833485608804132&z=5.

Figure 2 provides a summary conceptual visualization of CDI production as detailed in this Section, and in Supplementary Information B, we describe the step-wise process. Figure 3 is an example of publicly available CDI outputs from Morocco.

### CDI information dissemination using a web interface

**Figure 4:**
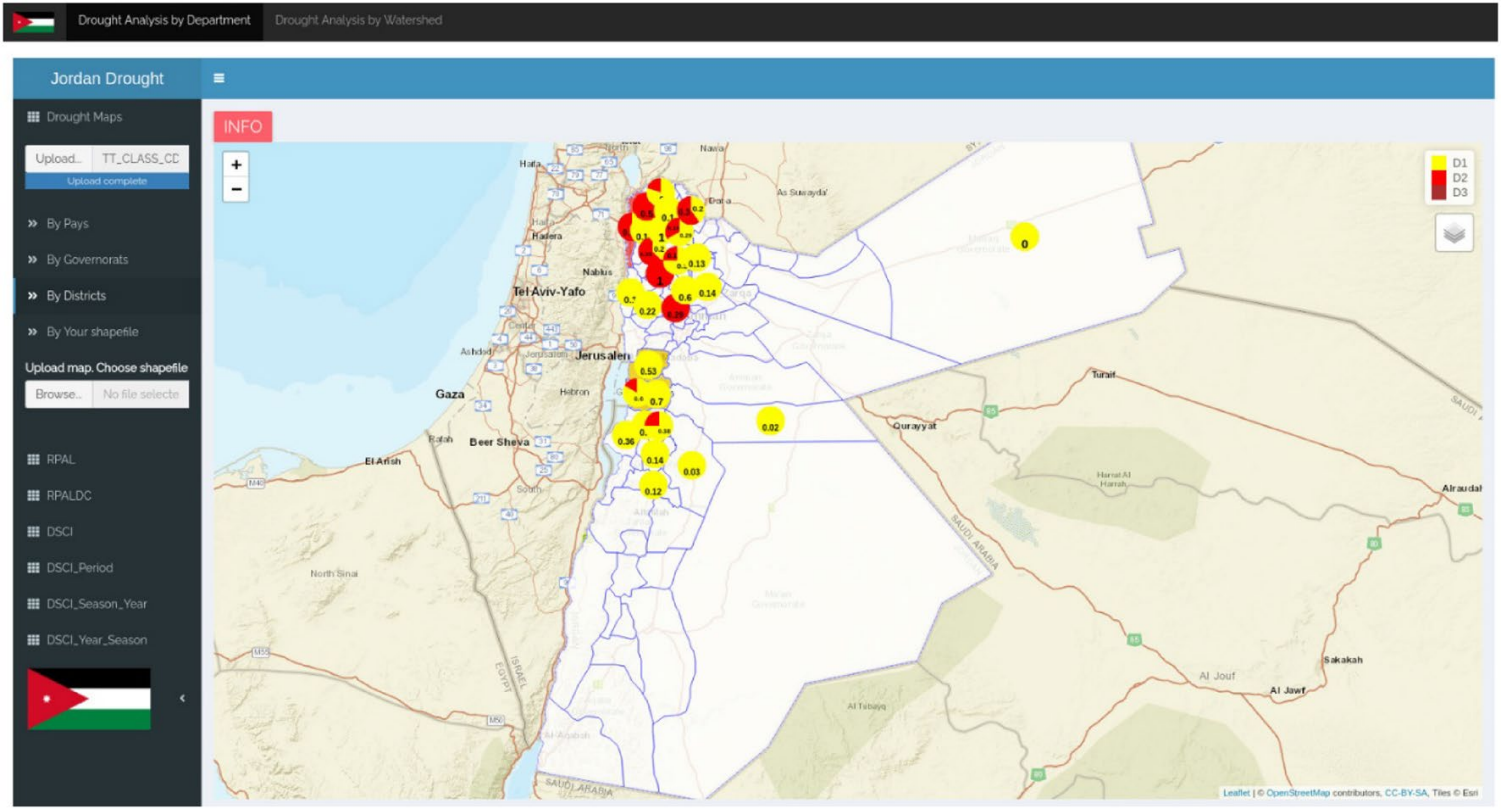
Web interface in Jordan showing sub-district level drought statistics (see Supplementary Information E for more detail on the web interface. Note that all maps in this article provide an overview of relevant drought monitoring domains, and they are neither a political statement nor a reflection of the authors’ position regarding the delineation of each country.

In Jordan and Morocco, CDI outputs are incorporated into a web interface (*accessibility*) that enables various temporal and spatial aggregations of CDI data. This simplifies analysis and comparison of drought events, and it supports ongoing CDI developments (*salience*). For example, the web interface can be used to calculate a ‘cumulative seasonal CDI’ to evaluate drought conditions in specific areas over an agricultural season or year^14^ rather than monthly, and it can calculate the Drought Severity and Coverage Index (DSCI;^47^) at various scales. Figure 4 shows a web interface screenshot of sub-district level DSCI statistics in Jordan of the type that ministry officials produce and use to support decision-making, and Supplementary Information E is example guidance for officials on use of the web interface.

## CDI validation findings and iterative refinements to the CDI

### Overview

In Stages 1 and 2, we evaluated the data used to produce CDI input indices, those CDI input indices themselves, and the integrated CDI in each country. This included:

- Qualitative assessment of CDI performance with national government stakeholders (during all Stages);
- Comparison with available observation data (primarily for precipitation);
- Assessment of index and/or CDI performance as a function of land cover and use as well as relationship with cereals production; and
- Semi-quantitative assessment of CDI performance with local drought impact reporters.

In this Section, we describe validation results in more detail and how they, and stakeholder constraints and needs, influenced CDI refinements and use over time. Further we describe in categorical terms the refinements made to the CDI and briefly the effect of those changes.

Table C1 summarizes all the validation assessments undertaken, their results, and subsequent CDI refinements (or considerations for future refinements) in each country. Additional results from this Section are shown in Supplementary Information C and D.

### Validation assessments and iterative refinements

#### Qualitative assessment of Stage 1 and 2 CDI

Stakeholders and the project team qualitatively assessed CDI outputs in all Stages in all countries. This happened in meetings and workshops that included both technical and policy staff from multiple government agencies (At least from meteorological departments and ministries with remits in agriculture and water; and Jobbins et al.^6^ describes the governance arrangements of the relevant inter-agency committees.) Participants used their expert opinion to assess CDI outputs from various years and seasons—particularly those with noted wet or dry tendencies and reported impacts^48,49^—and considering the results of analyses shown in this Section.

In Stages 1 and 2, stakeholders consistently noted concerns with CDI outputs’ spatial and temporal inconsistency. Firstly, missing data for input indices affected by cloud cover meant that the CDI outputs had missing monthly values for some pixels. This was especially problematic in mountainous areas of the project countries that are major sources of surface water runoff and groundwater recharge. Secondly, they described outputs as “noisy” because CDI classes varied significantly within relatively homogeneous sub-basins (i.e., “extreme drought” pixels adjacent to “moderately wet” pixels) or shifted between classes frequently across monthly time-steps. They considered this noise to be especially problematic in significant agricultural basins and rangelands zones.

Given the CDI’s intended spatial (sub-basin) and temporal (monthly) scales of assessment, this noise does not align with empirical knowledge of the relevant socio-environmental systems (*credibility, legitimacy*), though at finer spatial scales, this type of variation is expected due to micro-climates.

Through visual assessment of data outputs, we determined the main causes of noise were a.) the presence of clouds in the LST and NDVI products from MODIS (see Figures A2, A3, and A5), and b.) the short baseline period and therefore small population for percentile ranking of outputs. These findings led us to develop and apply—for all countries—the cloud masking and gap-filling methods for diurnal LST and NDVI (see sections "Novel application of harmonic analysis and Fourier transformations for cloud masking and gap-filling with data fusion" and "Improving NDVI outputs using Savitzky-Golay filters"), as well as the rolling window approach (2.3).

**Figure 5:**
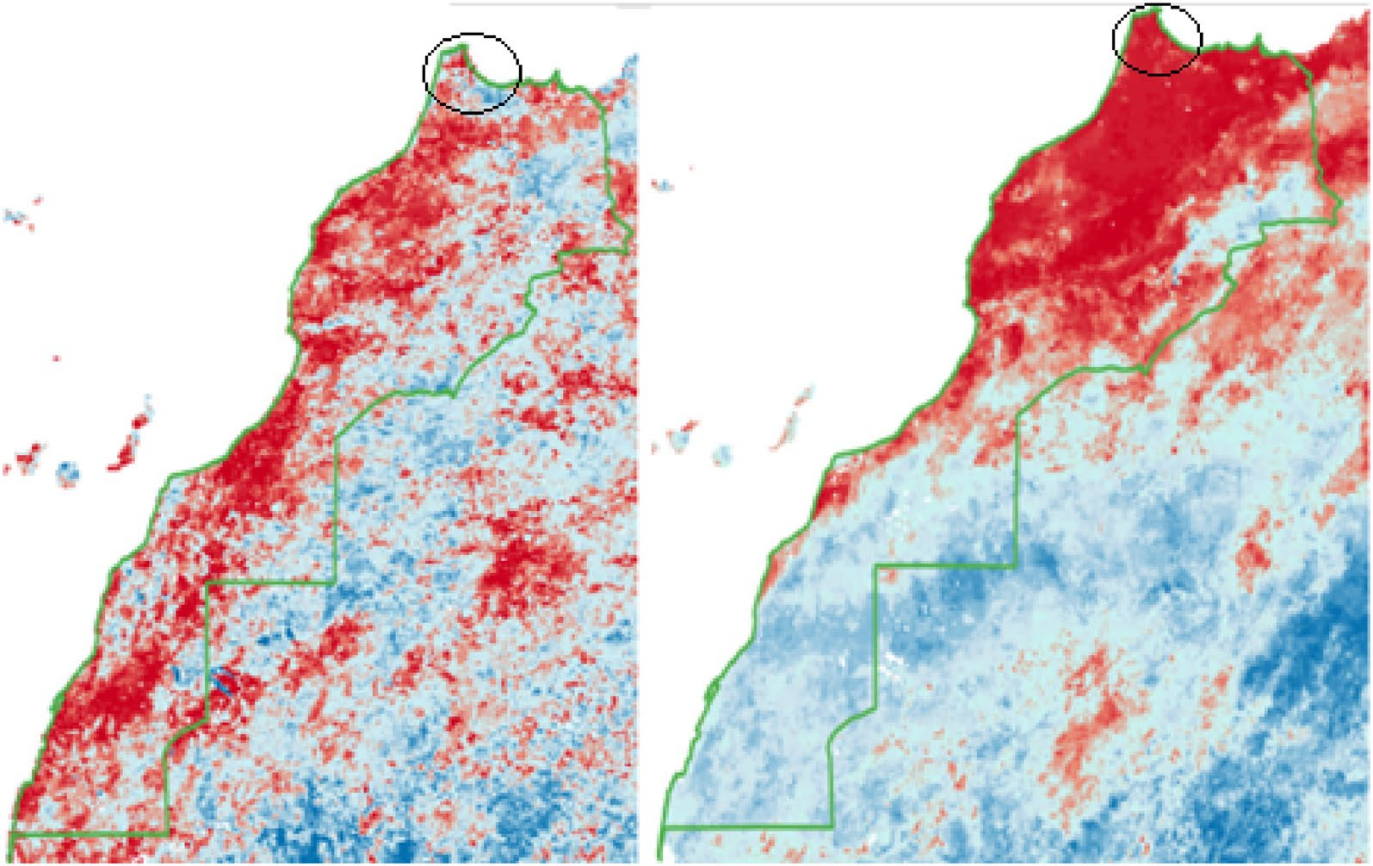
Comparison of Stage 1 CDI (left) and Stage 3 CDI (right) from Morocco for the month of January 2016. Map colors indicate drought classes ranging from dark red being exceptional drought to dark blue being exceptional wet, and the Tangiers and Rif area circled.

#### Iterative development of precipitation inputs

In Stage 1, we used CHIRPS final precipitation data to calculate SPI-3. We also investigated the use of IMERG data. IMERG has the full time-series available almost immediately after the end of the month as well as higher frequency of measurement. Respectively, this improves the timeliness of CDI production (*salience)* and statistical ranking (c*redibility*).

In Stage 2, we produced SPI-3 using IMERG data. We had concluded from a relatively cursory review that IMERG compared adequately with ground-based measurements from rain gauges and weather radar (*credibility*;^50–55^). However, subsequent analysis of observed precipitation data (see Table 2; Supplementary Information D) showed that CHIRPS’ final product—and in some cases even the preliminary product (which is usually available at the same time as IMERG)—outperforms IMERG in most areas of the countries.

**Table 2:** - Comparison of observation station precipitation data to CHIRPS and IMERG satellite products for Jordan, Lebanon, and Tunisia.

Country	No. of stations	Data range	Summary statistic	Correlation to observed		RMSE to observed	
				CHIRPS	IMERG	CHIRPS	IMERG
Jordan	25	Jan 2001-Apr 2021 (24); Jan 2005–Apr 2021 (1)	Mean	0.78	0.59	17.21	26.72
			Median	0.86	0.6	11.96	21.73
			Maximum	0.93	0.79	42.91	58.24
			Minimum	0.4	0.18	4.41	11.06
			Standard deviation	0.15	0.13	11.75	14.76
Lebanon	23	Highly variable between 2001 and 2018	Mean	0.81	0.64	55.40	82.92
			Median	0.84	0.67	52.75	75.03
			Maximum	0.96	0.8	109.12	164.02
			Minimum	0.27	0.21	23.16	49.06
			Standard deviation	0.15	0.15	20.34	26.70
Tunisia	15	Jun 2000–Dec 2020	Mean	0.83	0.54	19.72	75.23
			Median	0.83	0.55	18.52	67.79
			Maximum	0.91	0.66	36.14	122.79
			Minimum	0.72	0.36	10.43	26.88
			Standard deviation	0.062	0.094	6.62	30.36

National stakeholders then considered trade-offs related to products’ accuracy (geographical and precipitation intensity), spatial resolution, timeliness of CDI production, and model complexity, and each national agency chose which input to use for the operational CDI as shown in Table 1 (*credibility, salience, accessibility*).

In Morocco, work on seasonal forecasting^14^ and the presence of relatively strong technical capabilities and computing capacity, enabled straightforward application of CNN models to improve the accuracy of CHIRPS’ preliminary product (see Supplementary Information A). This improved the timeliness of CDI production (*salience)* while minimizing loss in accuracy (*credibility).* In Jordan, officials opted to retain the use of IMERG because it proved more accurate in some *badia* semi-arid and arid regions where communities have high vulnerability to drought impacts (*credibility, legitimacy;*^12,48^. In Lebanon and Tunisia, modelling simplicity and information accuracy trumped the timeliness of production, and so they chose the CHIRPS final product (*accessibility, credibility)*.

#### Assessment of relationship between CDI and rainfed agricultural systems’ production

In Stage 2, we quantitatively assessed the relationship between cereals production and yield data to CDI input indices and the integrated CDI in Jordan and Morocco (*legitimacy*). Results indicated that the CDI performed better than precipitation indices alone, though the relationship was complex due to the timing of drought stress and the specific agricultural effect (see Supplementary Information C and Tables C2 and C3). These analyses were not repeated in Stage 3 to compare results statistically.

These results supported stakeholders’ use of the CDI outputs for drought impact management through Drought Action Plan development (*legitimacy, salience*;^6^. They also informed the subsequent development of regression relationships between temporally and spatially aggregated CDI results and annual cereal production values^14^ as well as semi-quantitative evaluations described next.

### Drought impact reporters’ semi-quantitative assessment and policy application pilots (Stage 3)

In Stage 2, we undertook semi-quantitative assessment of Tunisian local government officials’ assessment of CDI outputs. In Stage 3, we assessed drought impact reporters’ perceptions of CDI maps generally (various stakeholders, Tafilah Governorate, Jordan), and specifically in relation to rangelands (Moroccan local agriculture officials covering 27 rangelands areas—over 1.9 million ha, 90% of the officially recognized total).

Unfortunately, data quality from responses in Tunisia precluded structured analysis (response rate and location discrepancies), but the limited findings were consistent about CDI noise described in section "Qualitative assessment of Stage 1 and 2 CDI" and were also useful to consider highly local distribution of drought impacts and its relevance for policy decision-makers.

The Jordanian and Moroccan evaluations (results shown in full in^12,14^) supported CDI validation as well as piloting policy applications. In Jordan, drought reporters’ perceptions generally aligned with the CDI output in terms of temporal and spatial coverage as well as intensity; they found that the CHIRPS precipitation product over-estimated precipitation in Tafilah compared to IMERG and station data. In Morocco, there was strong alignment between CDI information and expert estimation of drought location (average 74%) and severity (average 71%), with lowest accuracies in forested and snow-affected areas.

In Jordan, this validation informed the choice of precipitation index inputs for the CDI as well as disbursal of government funds (*Takaful fund)* for drought-impacted smallholder farmers; in Morocco, it tested the CDI’s viability to support implementation of Rangelands Law 113–13 and provided evidence to inform potential future CDI modifications for that purpose (e.g., land cover masks).

### Programming refinements to ease of CDI production

Stakeholders consistently noted the need to minimize the complexity and computing requirements for CDI production because agency officials had to be able to produce it independently^3^. This took some trial and error.

During Stages 1 and 2, the CDI production process was cumbersome and national agencies did not have the capacity to do the advanced modelling regularly. Therefore, project team staff provided SMA and diurnal LST inputs to national agencies who then integrated them into the CDI.

The initial programming framework was in Linux. However, the lead agencies had few people skilled in use of Linux. During Stages 1 and 2, as a first attempt to simplify the process, we migrated the programming framework to Windows. Unfortunately, frequent Windows updates were highly problematic because they led to issues such as code libraries being corrupted or removed on a regular basis.

In response, in Stage 3, we returned to Linux and focused instead on ensuring the programming framework linked all CDI production components effectively, especially the necessary LIS and Noah-MP model components. Likewise, we developed scripts to make most of the CDI production steps nearly fully automated. The most important aspect to simplify (because of the underlying coding complexity) was to make the Noah-MP model link to and be interoperable with the CDI model. Also, we supported installation and use of dedicated servers for data processing and modelling, as well as ongoing training for agency staff to use the LIS and CDI modelling frameworks.

### Objectives of CDI refinements and description of refinements in categorical terms

In summary, refinements made to the CDI system between Stages 1 and 3 aimed to:

- ensure the CDI outputs cover the entire domain each month (*credibility*);
- reduce CDI output noise and improve spatio-temporal coherence (*credibility and legitimacy*); and
- enable national agencies to produce the CDI autonomously, quickly and regularly, and relatively easily (*salience*)

In categorical terms, the CDI validation efforts informed subsequent refinements in accordance with Rykiel’s^18^ schema:

- Model re-calibration through changes in parameter values;
- Structural changes to the model; and
- Restricting the model to a smaller domain.

CDI modelling refinements from the Stage 1 to Stage 3 CDI included the following (primarily *credibility*; secondly *salience* and *legitimacy*):

- Change in input datasets for SPI-3: from CHIRPS final to other products in some countries (see Table 1);
- Additional pre-processing of input data, and change in input dataset for diurnal LST: use of harmonic analysis and gap-filling (see section "Novel application of harmonic analysis and Fourier transformations for cloud masking and gap-filling with data fusion");
- Additional pre-processing of input data for NDVI: use of Savitsky-Golay filters (see section "Improving NDVI outputs using Savitzky-Golay filters"); and
- Modification of the CDI ranking and calculation procedures: use of sliding windows (see section "CDI integration and ranking using sliding windows").

Additionally, to accommodate the end of the MODIS mission, we modified the CDI framework to use either eMODIS or VIIRS input data through the “creation” of VIIRS data for 2000–2012 using eMODIS data and applying geometric mean regression^56^ to determine regression coefficients at the pixel level (*accessibility*).

### Assessing effects of CDI refinements

**Figure 6:**
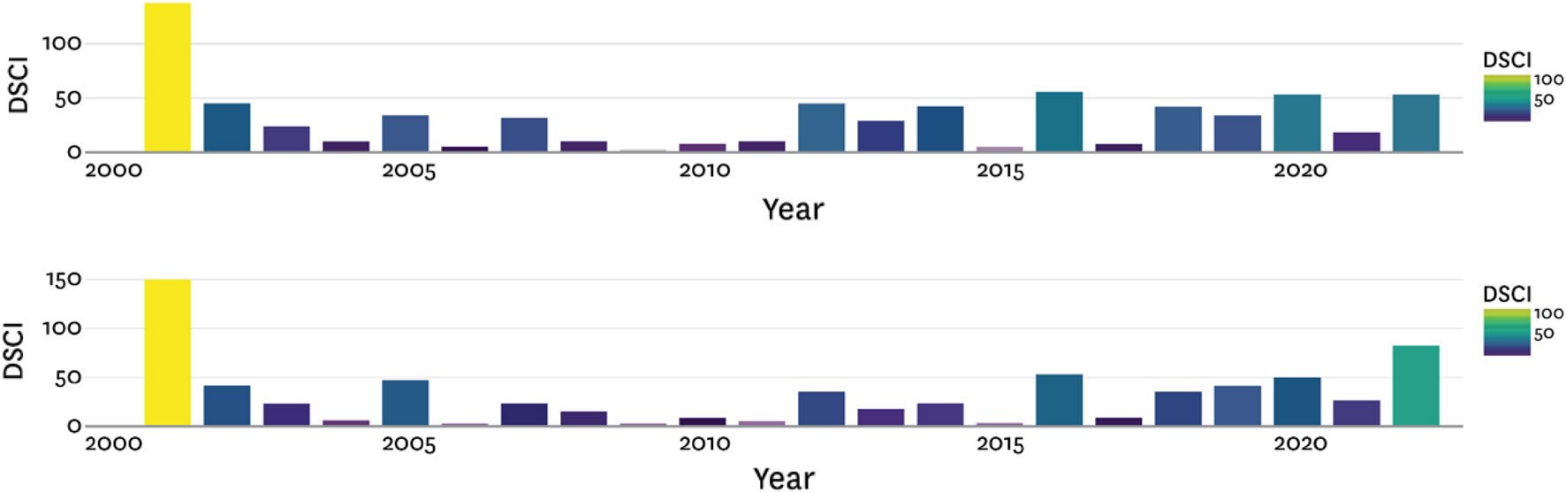
The web interface in Morocco showing the 2000–2022 annual DSCI calculated using the Stage 2 CDI (top) and Stage 3 CDI as described in Table 1 (bottom).

We presented CDI outputs from various Stages side by side for expert officials to evaluate. This included (a) focused consideration of wet and dry months and years they knew well (*credibility, legitimacy;* example in Fig. 5) and (b) information from the entire period (2000-present) presented in relation to CDI monthly values as well as annual aggregations (example in Fig. 6;^57^).

They concluded from these assessments, in conjunction with drought impact reporters’ results (section "Drought impact reporters’ semi-quantitative assessment and policy application pilots (Stage 3)"), that refinements had substantial positive effects on CDI outputs, especially in relation to locational accuracy and CDI noise in a manner concordant with expectations.

For example, in Fig. 5, the Stage 1 CDI showed severe drought categories (dark red) interspersed with relatively humid categories (light blue). In contrast, the Stage 3 CDI was more consistent regionally, and the localized variation was less extreme. Also, the Stage 1 CDI miscategorized the coastal Tangiers and Rif area as relatively humid (the Stage 3 CDI had it as severely dry), when in reality there were significant drought impacts there^58^.

Likewise, Fig. 6 shows the Stage 2 CDI (using IMERG and without the modelling improvements) and Stage 3 CDI outputs aggregated annually using the DSCI. Stakeholders considered the Stage 3 CDI outputs to reflect inter-annual progression of dry years in the late 2010s and early 2020s more accurately per their knowledge of meteorological, agricultural, and hydrological conditions in those years.

## Discussion and conclusions

### Considering trade-offs and constraints in CDI development

At the outset, we faced three critical questions framing CDI development:

1. What type of process or decision-making should the monitoring information support^59^;
2. Which socio-environmental system(s) should be the focus of monitoring^60^; and
3. How to develop the CDI so that agencies can and do continue to produce it^16^.

Stakeholder needs assessments^3,4^ and subsequent engagement for Drought Action Plan development enabled articulation of CDI requirements, which largely answer questions one and two: the CDI should provide technical “triggers” for drought management actions focused on rainfed agriculture^6^. Further, considerations of which specific areas were prioritized for monitoring guided subsequent modifications. For example, in Jordan, the desire for most accurate results in *badia* and certain arid areas guided choices about SPI-3 data inputs and in Morocco, needs for rangelands monitoring guide near-term potential improvements.

Most of the research presented in this paper relates primarily to question three: in CDI development, our core focus was balancing the need for a relatively simple modelling system that met the credibility, salience, legitimacy, and accessibility requirements for political decision-making. This was a critical consideration for CDI development.

For instance, as shown in Table C1, validation studies proposed modifications to the CDI structure such as variable input weights per agro-ecological zone or land cover class. However, government officials in all countries chose not to pursue that route for multiple reasons:

- The simplicity of CDI calculation, production, and interpretation is a paramount feature for its *salience*, and adding such complexity would undermine those core characteristics^61^;
- They took statistical and ontological uncertainties^62,63^ into consideration: the additional scarcity (and in some cases absence) of observation data in each classification zone meant that outputs of a more complex and spatially precise CDI could not be evaluated as rigorously using observation data (*credibility)*, and
- Given that ground-truthing was critical for legitimacy, any changes that would preclude agencies’ ability to validate the outputs with observation data would lead to outputs being perceived as less *credible* and *legitimate*^61^.

Therefore, rather than focusing energy and resources on geographically determined model refinement at this stage, government agencies prioritized general modelling improvements and building user/stakeholder engagement mechanisms including officials in the regions to provide ongoing quantitative and qualitative feedback on the CDI like that provided by drought impact reporters (section "Drought impact reporters’ semi-quantitative assessment and policy application pilots (Stage 3)").

### Value and challenge of using a common regional modelling framework

The MENAdrought project always intended to develop drought monitoring systems for national application. The fact that stakeholders in all countries prioritized monitoring associated with rainfed cereals impacts enabled us to start from a common modelling framework and tailor the system to fit individual countries’ needs while replicating improvements across countries efficiently. This supports replicability of the modelling system in other countries to support the World Meteorological Organization’s objective to cover all people with early warning systems by 2030^64^. Indeed, already the system has been replicated with government agencies in Tunisia^14^ and used in Georgia^65^. It also enables regional approaches to training and peer learning, including the possibility for regional centres of excellence.

At the national level, having a common modelling framework across agencies is key because it requires involved agencies to agree on the underlying objectives and purposes of drought monitoring, and by implication eventual drought definitions. This supports cross-government coordination in both drought monitoring and management responses.

Still, challenges with employing a common modelling framework are rife. In particular, data-sharing and associated coordination within countries and at the regional level continue to be primary barriers. Also, involved agencies have different remits and underlying goals, and so reaching common ground takes time and effort of which output validation is a core component.

### Considering model validation in an operational policy context

Our findings show that multiple types of validation were necessary for stakeholder acceptance of the CDI. Quantitative and semi-quantitative validation were critical for stakeholder perceptions of the CDI’s *salience* and *credibility*^15^ despite the dearth of observation data. In addition, much like for the development of the U.S. Drought Monitor^7,66,67^, qualitative application of expert opinion and subjective knowledge—elicited in multiple manners—were also critical for stakeholder perceptions of its *credibility* and *legitimacy*.

This is likely because the process enabled the application of multiple knowledge types and fostered (a) transparency in the product’s development, (b) officials’ perception that the CDI is credible as we have improved it over time, and, (c) heightened trust in its developers and purveyors through ongoing collaboration and problem-solving^2,16^.

Agencies’ continued production of the CDI by their own staff on their own servers, its incorporation in Drought Action Plans, and its use to target relief efforts and otherwise support consequential decision-making indicate that government stakeholders consider it valid^6,59^. Also, agencies have identified future paths (for example, use of land cover masks or crop maps) to increase the CDI’s *credibility* overall and *salience* to support specific types of decision-making, though these would increase CDI production complexity.

These results strongly reinforce findings elsewhere (e.g.,^10^) that development of environmental monitoring tools for policy application benefits from sequential and iterative development as well as robust interaction between producers of the tools and their end-users. Further, they show potential routes to consider, develop, and test robustly the scientific tools for policy application that governments across the Global South increasingly use, and that international institutions and donors incentivize them to use through climate change adaptation funding mechanisms^64^.

### Supplementary Information

Supplementary Information 1.

Supplementary Information 2.

## Data Availability

The datasets generated and/or analysed during the current study are not publicly available due to the fact that they are produced by the relevant government agencies but are available from the corresponding author on reasonable request.
