# Supplementary material for: Development of a composite drought indicator for operational drought monitoring in the MENA region
Source: Sci Rep. 2024 Mar 5;14:5414. doi: 10.1038/s41598-024-55626-0 (PMC10914844; doi:10.1038/s41598-024-55626-0)
Supplement: Supplementary file 1 — Supplementary Information 1. [file 41598_2024_55626_MOESM1_ESM.docx]

# Supplementary Information A – Additional information on Stage 3 CDI input index production

## Novel application of harmonic analysis and Fourier transformations for cloud masking and data fusion for gap-filling related to diurnal LST

Note that this content repeats some material from Section 2.2.2, but it provides more fulsome detail and explanation as well as pseudocode for the process.

In relation to identifying cloud presence, we calculate values for the HANTS-derived time series (T_s_^hants^) as follows in Equation A1. A temporal sequence of N observations *y_i_, i= 1 to N* can be described by a Fourier series as:

**Equation A1:** $y_{i}=a_{0}+\sum_{j=1}^{M} \left( a_{j}cos\left( w_{j}t_{i}-Фi \right) \right)$

Where t_i_ is the time at which the i^th^ sample was taken, M is the number of frequencies of the Fourier series (M <= N), ${a_{j},w}_{j}$ and $Фi$ are respectively the amplitude, the frequency and the phase of the j^th^ harmonic term in the Fourier series.

As an example, for Figure A1 below, HANTS was applied on 1-year daily MOD11A1 data from 2015. It has a frequency M=1 and a number of observations N=365. T_s_ values that deviated more than 4°C from the T_s_^hants^ were flagged as cloud affected (T_s_ – T_s_^hants^ < -4°C) and subsequently removed from the clean data set. These pixels were later replaced by updated estimates of T_s_ as described in Section 2 and the algorithm below.


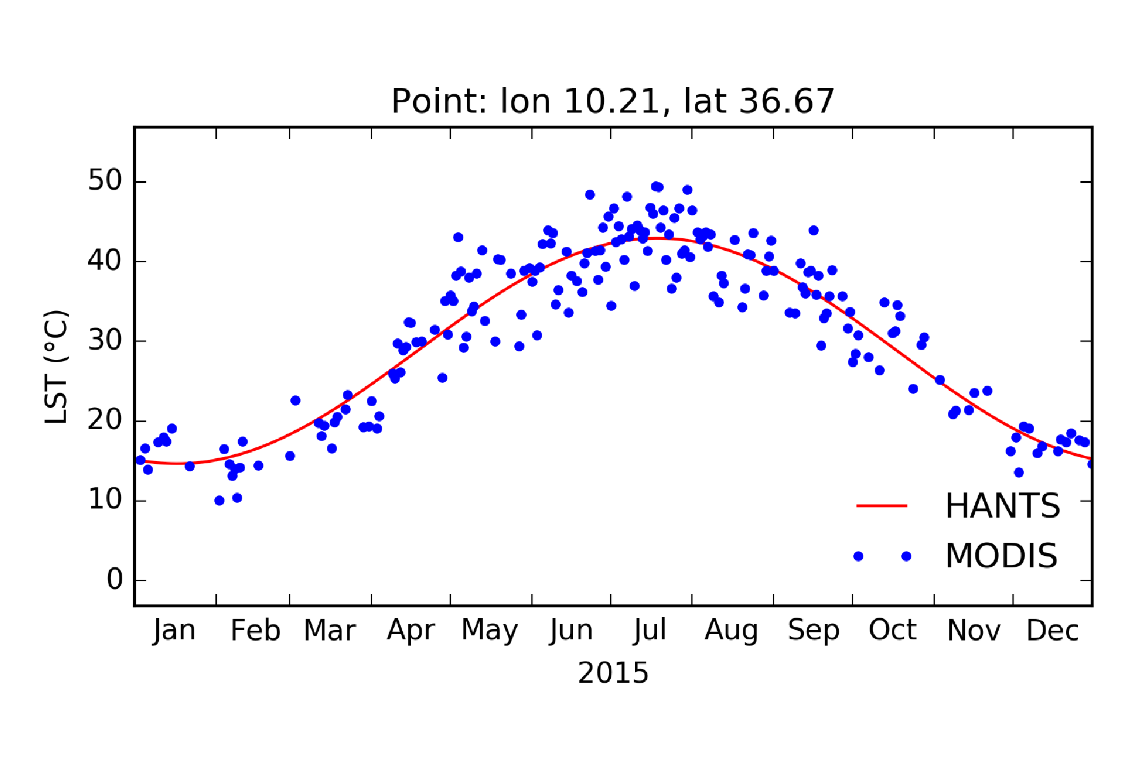


**Figure A1:** Comparison of daily LST from MODIS (blue dots) under clear sky conditions. The smoothed T_s_ time series using HANTS (red line) (T_s_^_hants^) with a frequency equal to 1. One pixel in Lebanon taken for the sake of demonstration.

| Psuedocode for MODIS data cloud gap-filling |
| --- |
| 1: for each month do  3: Produce smoothed time series (T_s_^hants^) from MODIS land surface temperature (Ts)  4: if T_s_ - T_s_^hants^ > 4°C then  5: Flag as cloud affected  6: end if  2: for each time step do  7: Generate “daily perturbation” model (dTobs) for MODIS observed Ts and smoothed Tshants  8: dT_obs_ = T_s_ – T_s_^hants^  9: Simulate LST values (T_s_^model^) using Noah-MP and produce a smoothed time series (T_s_^hants_model^)  10: Generate “daily perturbation” model (dT_model_) of the simulated values  11: dT_model_ = T_s_ – T_s_^hants_model^  12: Estimate dT_obs_ for clear and cloudy using a simple linear regression model.  13: dT_obs_ = a * dT_model_ + b  14: if flagged as cloud-affected then  15: Correct the smoothed T_s_^hants^ and reconstruct temperature data (T_s_^reconst^) for cloudy days  16: T_s_^reconst^ = T_s_^hants^ + a * dT_obs_ + b  17: end if  18: end do  19: end cloud gap-filling |

In the presence of clouds, a non-realistically large difference between the T_s_ value and its smoothed time-series of T_s_^hants^ arises. The new cloud mask based on the threshold of -4°C performed well (Figure 2) and detected cloudy pixels at the edges of the clouded region that were missed by the original MODIS cloud mask. An example is shown in Figure A2.


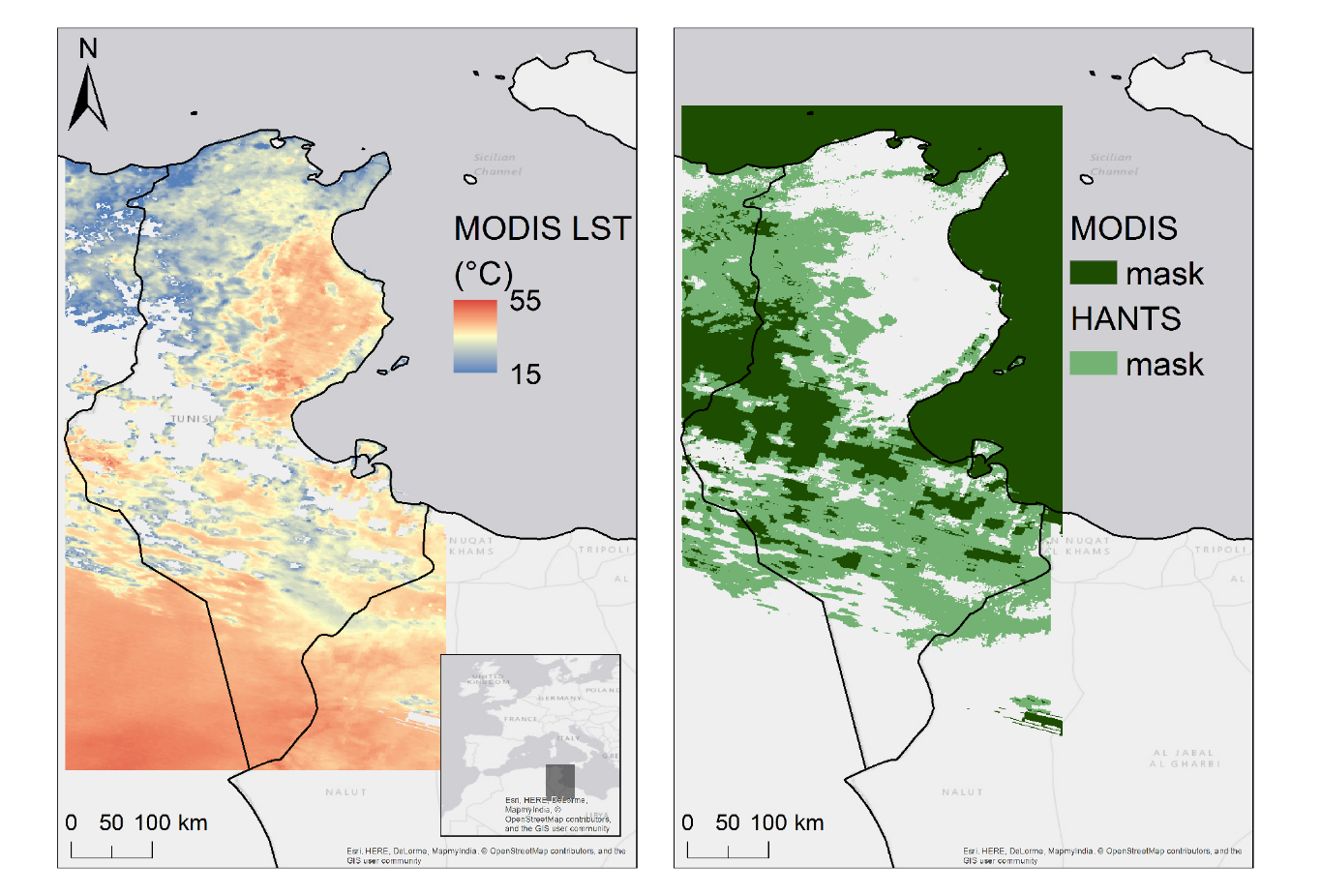


Figure A2 – Cloud mask derived from harmonic analysis. Left image shows MODIS LST of 1 June 2015 over Tunisia. Right figure shows MODIS-derived cloud mask and sea (dark green) and HANTS-derived cloud mask.

The gap-filling method was applied after producing the cloud mask. To evaluate the approach, we assessed the diurnal LST anomaly in Lebanon resulting from the quality-controlled and reconstructed (using the gap-filling method) data of the same day. Lebanon is a good example because it has frequent, widespread winter cloud-cover and highly variable topography. Figure A2 shows the difference between the reconstructed and quality-controlled MODIS LST data for both day and night.


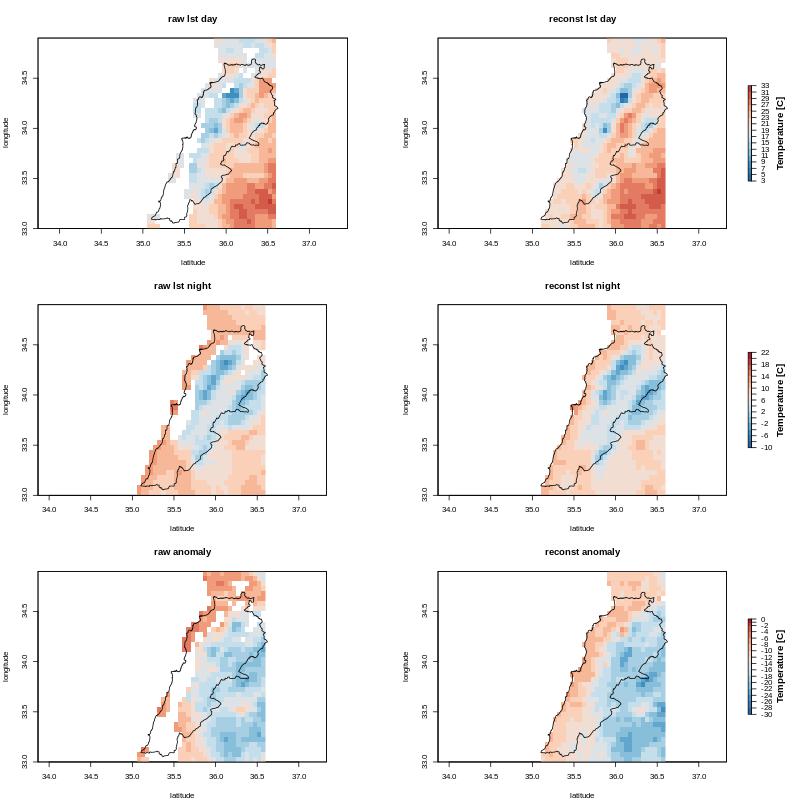


**Figure A3** – Comparison of quality-controlled MODIS to reconstructed diurnal LST (5km x 5 km). Quality-controlled MODIS day (top-left), night (middle-left), and diurnal amplitude of LST (bottom-left); reconstructed day (top-right), night (middle-right), and diurnal amplitude of LST (bottom-right). Note the large areas of south-western and western Lebanon with no values in the bottom left.

Further, we calculated the RMSE of the observed and reconstructed diurnal LST over clear-sky pixels for all of 2018. We obtained an RMSE value of 0.4°C for the whole domain (42 x 40 pixels), which indicates a very accurate simulation of the day-night LST amplitude. Figure A3 below shows the geographical distribution of average and absolute error over the whole domain for all clear-sky pixels in 2018.

The error tends to be slightly higher in mountainous areas and slightly lower in the plains. It generally does not exceed 0.5°C, and we consider an error of 0.5°C to be acceptable (remains *credible)* for operational use within the drought monitoring system.


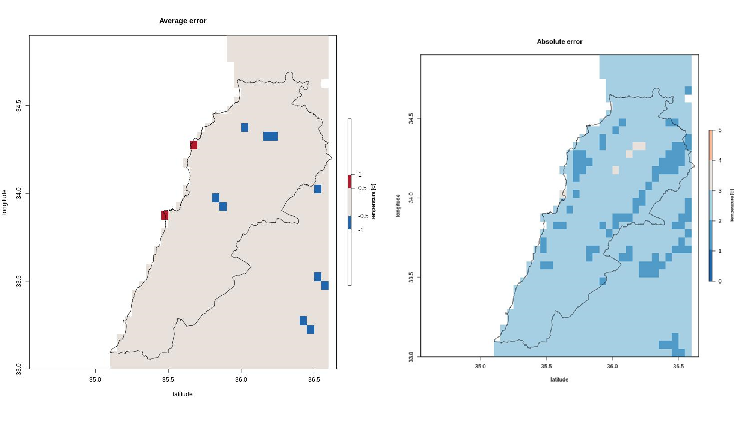


**Figure A4 –** Average (left) and absolute (right) error of quality-controlled MODIS compared to reconstructed diurnal LST for clear-sky pixels (5km x 5 km) using all data from clear-sky pixels in 2018.

Overall, the reconstructed diurnal LST follows observed diurnal LST spatial patterns and magnitude very closely. The results provide reassurance that the model reconstruction for gap-filling matches the observed (remotely sensed) data input to the CDI. Additionally, it significantly expands the number of pixels for which the daily values (and therefore the whole index each month) are calculated, which expands the product’s salience.

## Development and use of Convolutional Neural Network models to improve CHIRPS preliminary data for Morocco

Section 3.2.2 describes the process and assessments undertaken to arrive at Stage 3 CDI precipitation input, and Table 1 details production of SPI-3 except for the use of convolutional neural networks (CNN) in Morocco to estimate the CHIRPS final product using CHIRPS preliminary product data and IMERG data as the predictors. Bergaoui et al. (2022) describe the development of these CNN models and associated regionalization more fully, and here we summarize the process.

We developed 12 month-specific CNN models. They take CHIRPS preliminary data and near-real-time IMERG data over the Moroccan domain as the predictors for CHIRPS final data. The loss function of the CNN model, re-written by the authors, uses a regionalization technique, which consists in identifying coherent regions based on their specific rainfall regime [68]. This maximizes clustering and minimizes differences within regions by instructing the model to “focus” improvement of the loss function on those areas of interest [69]. This approach leads to differentiation in model application for more humid and more arid areas to reduce the errors in each. In this application, Morocco had 7 climate regions, and we trained the CNN models using CHIRPS final data from the 1982-2014 period.

NDVI
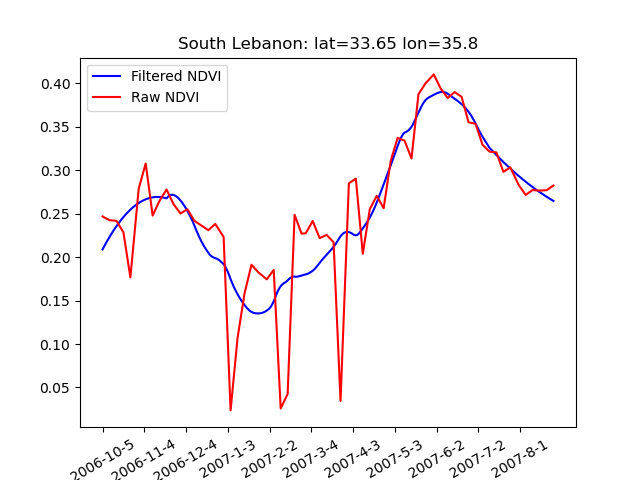


**Figure A5 – Example NDVI time-series for a pixel in Lebanon over the hydrological year of 2006-2007. The red line is quality-controlled eMODIS data and the blue line is the filtered time-series.**

# Supplementary Information B – Step-wise description of monthly CDI production process

Following LIS and Noah-MP model installation and parameterization (see 2.2.1), agencies now undertake the following process to produce, within 10 days of the beginning of the month, the CDI for the preceding month. Almost all of this happens through one program coded in Python.

**Step 1:** CDI input index data are downloaded from the websites at the frequency described in Table 1. Then they pre-process the data in Steps 2-5.

**Step 2:** GDAS forcings and the Noah-MP parameters (land cover, soil texture, and topographic characteristics) are input to the LIS model, which produces the root zone soil moisture and LST data on an hourly time-step (see 2.2.1).

**Step 3:** MODIS day and night LST data are inputs to the HANTS model, which filters out cloud-affected pixels.

**Step 4:** MODIS day and night LST data, and hourly LST data from the LIS model (from Step 2), are inputs to the HANTS model and gap-filling procedure, which produces cloud-free 1km x 1km observed day and night LST data (see 2.2.2).

**Step 5:** eMODIS NDVI data are input to the Savitzky-Golay filter, which a.) filters out cloud-affected pixels and b.) produces smoothed daily data for all pixels (including cloud-affected; see 2.2.3).

Following this data pre-processing, we calculate the monthly CDI map as follows in Steps 6-11.

**Step 6:** Aggregate root zone soil moisture data (outputs from Step 2) to daily data, then apply the sliding window with 2-days window to produce 6 values for the current month, and then rank them to percentiles in relation to values from the entire 20-year+ period.

**Step 7:** Calculate the day-night land surface temperature amplitude (outputs of Step 4), and then apply the same sliding window and ranking as for Step 6

**Step 8:** Aggregate the daily filtered NDVI data (outputs of Step 5), then apply the same sliding window and ranking procedures as for Steps 6 and 7.

**Step 9:**Apply the same sliding window on the relevant precipitation data (per Table 1), then calculate the 3 months SPI-3 on each of the 6 values, and then rank them into percentiles.

**Step 10:** Combine the six representations of the four input indices into the six generated CDI values for the current month, and rank them into percentiles.

**Step 11:** CDI_6_ is reported according to the different classes of drought shown in Section 2.4.

# Supplementary Information C - Additional validation results

Table C1 – Summary of CDI validation analyses, results, and refinements made (or to be considered in the future)

| Country | Indicator | Validation analyses | Results | Refinements made or considered following validation |
| --- | --- | --- | --- | --- |
| Jordan | SPI-3 | Comparison of CHIRPS final and IMERG precipitation data to long-term (1980-2017) station data (site-specific and spatial interpolation from 155 stations) | See Tables 2, D1, and D4. CHIRPS has good but variable R^2^ – best performance was during rainy season in wetter regions (e.g. Irbid R^2^=0.69); worst performance was in desert regions. IMERG performed well only in *badia* and desert areas. | Shift to IMERG (see 3.2.2 for rationale) and proposed consideration of shift SPI-2 (not implemented). |
|  | SMA | Compared model output soil moisture to 2015-2016 soil moisture data from 11 ground stations in and near Irbid Governorate (northern highlands). | Low R^2^ (0.37) except for one site with clay soils, which had higher soil water holding capacity (closer to parameter value in LIS model). However, major difference in scale (1km x 1km model output versus individual soil sensor) introduces uncertainty. | For all countries’ CDIs: Introduced the irrigation scheme in Noah-MP model implementation and subsequently removed it and introduced the  dynamic phenology scheme in Noah-MP model implementation (see 2.2.1) |
|  | CDI | 1. Evaluated relationship of each indicator and overall CDI (and for multiple time-steps of each) to cereals (wheat and barley) yields and production per governorate [70] 2. Compared to an ‘observation-based CDI’ [70, 71] 3. Qualitative evaluation of CDI’s performance in reflecting past droughts – workshops with key stakeholders 4. Semi-quantitative evaluation of CDI’s performance in reflecting the 2020-2021 drought in Tafilah Governorate [12] | 1. See Table C2. Correlation between CDI and cereals production was higher than precipitation indicators (p<0.05) and varied highly across regions; 2. Both products generally agreed and successfully identified trend (both drought and wet periods), drought intensity, and location; 3. Same as for point (2), and stakeholders provided recommendations for further improvements; 4. Drought reporters’ perceptions generally aligned with the CDI output in terms of temporal and spatial coverage as well as intensity; CHIRPS product found to over-estimate precipitation in Tafilah compared to IMERG and station data. | 1. Proposed to investigate shift in indicator time-steps and weightings to fit CDI to cereals production (neither implemented yet);   2. and 3. N/A  4. Decision to keep IMERG precipitation input due to better performance in *badia;* consideration of developing a *badia* and desert-specific CDI with different input index weightings (not implemented yet). |
|  |  |  |  |  |
| Lebanon | SPI-3 | Compare CHIRPS and IMERG to station data (2000-2016) from 3 sites in coastal, inland, and semi-arid areas. | See Tables 3 and D3. CHIRPS has very high R^2^ in coastal (0.86) and inland (0.90) areas; very low in semi-arid (0.26). | Stage 3 return to CHIRPS final product (see Section 3.2.2). |
|  | NDVI | Comparison of NDVI in agricultural, high elevation bare land (snow-affected), and mixed-evergreen forest at mid-altitudes | NDVI signal highly influenced by snow and cloud cover during the winter season, especially January to March. | For all countries’ CDIs: Improved cloud masking procedures and use of daily interpolation (see Section 2.2.3) |
|  | SMA | Comparison of CHIRPS data and soil moisture model output in semi-arid areas. | 1. Model output spatially displaced against other indicators (shifted eastwards); 2. SM over-estimated for inland areas, at times exceeding precipitation, but overall correlation suggests SM data was capturing the signal in dry/wet months. | As for Jordan |
|  | CDI | 1. Attempted to assess relationship between CDI and available national statistics on hydrology, water use, agriculture, and ecology 2. Evaluate correlation and temporal relationship between each input for 50 pixels covering major land cover/use types. 3. Compare CDI with FAO-produced agricultural stress index and precipitation percentiles | 1. The sparse temporally and spatially discontinuous available statistics precluded statistical analyses of correlation between CDI and the indicators. 2. High variability in inputs’ correlation and temporal relationship dependent on land and cloud cover; 3. For most years (except 2000 and 2001), the CDI was in agreement with the national climatological drought and the FAO’s Agricultural Stress Index (ASI). | 1. N/A 2. For all countries’ CDIs: introduced cloud detection and gap-filling procedures for diurnal LST (see Section 2.2.2); proposed to develop separate CDI input weightings based on land cover (not implemented) |
|  |  |  |  |  |
| Morocco | SPI-3 | Compare CHIRPS and IMERG to observation station data | See Tables 3 and D2. Results shown in Table D2 as described by [17, 72]. | Initial shift to IMERG, and subsequent shift to CHIRPS preliminary product and use of convolutional neural network models to “estimate” CHIRPS final product from it (See Supplementary Information A) |
|  | CDI | 1. Assess relationship between CDI (and its component inputs) and cereals production and yield anomaly for three agricultural years, each of which had a different drought typology. 2. Survey of regional technical experts to assess CDI performance and indicator relevance per region via AHP method [73]. 3. Semi-quantitative evaluation of CDI’s performance in reflecting drought effects on nearly 2 million hectares of rangelands with local rangelands monitoring expert officials [14] | 1. See Table C3. Results show clear concordance for most agro-ecological zones but not all, and/or for specific periods of the drought year but not all. 2. Survey results align with statistical analyses of concordance between CDI and agriculture statistics; indicator relevance informs re-weighting 3. Strong alignment between CDI and expert estimation of drought location (average 74%) and severity (average 71%) with lowest accuracies in forested and snow-affected areas. | 1 and 2: Proposed to investigate separate input weights for CDI per agro-ecological zone (not implemented yet)  3. Suggestions to mask specific land cover classes to improve CDI calculation for rangelands monitoring purposes (not yet implemented). |
|  |  |  |  |  |
| Tunisia | SPI-3 | Compare CHIRPS and IMERG data to monthly data from 326 ground stations | See Tables 3 and D5. CHIRPS more effective, and CHIRPS data most closely matched ground station data in the northern, more humid areas, and less closely down the north to south and west to east precipitation gradients. Matches were highest in the rainy season, particularly January – April. | Initial shift to IMERG, and subsequent return to CHIRPS final product. |
|  | NDVI | Assessed relationship between NDVI input and wheat yield in Tunisia from 2000-2018 using a cereal crop mask. | Wheat yield and MODIS NDVI are well-correlated, especially in the northern breadbasket. | As for other countries, improve cloud-masking and gap-filling |
|  | CDI | Semi-quantitative evaluation of CDI’s performance in reflecting drought effects on rainfed cereals and olives in 2017-2018 agricultural year | Relatively high monthly and regional variation in validators’ assessment of CDI outputs accuracy in relation to geographic distribution and intensity. | Proposed to investigate:  1. Variable CDI weighting per agroclimatic zone  2. Develop a cumulative CDI for the entire agricultural year (this was developed in Morocco – see [14])  3. Validation of diurnal LST with water balance model such as SWAT.  4. Mask irrigated areas and focus only on rainfed agriculture |

## Stage 2 validation analyses in Lebanon

In Lebanon, government stakeholders indicated that NDVI signal was good in areas with agriculture, pasture, and forest land covers. However, above mid-elevation mountain regions, the response was problematic over the winter season – particularly between January and March – due to cloud and snow cover (see Figure A3). This issue – also common to mountainous areas of Morocco and Jordan – suggested the need for cloud gap filling of the NDVI and diurnal LST data.

*CDI validation*

In Lebanon, we were only able to evaluate the CDI nationally in relation to precipitation observations from a very small number of meteorological stations and the FAO’s Global Information and Early Warning System (GIEWS) data: [http://www.fao.org/giews/earthobservation/country/index.jsp?lang=en&code=LBN#](http://www.fao.org/giews/earthobservation/country/index.jsp?lang=en&code=LBN). It performed favorably and only had significant disagreement in two years of the period, 2000 and 2001. In those years, the CDI showed higher drought stress than the observed precipitation ranking and the FAO product.

In relation to precipitation, such difference can be attributed to the scarcity of the national Lebanese meteorological observatory and the limited numbers of available stations. The FAO Agricultural Stress Index (ASI [74]) is based solely on NDVI data, which may explain some differences with the CDI.

## Stage 2 CDI validation in Jordan

In Jordan and Morocco, CDI input indices, and the integrated CDI, were evaluated against rainfed agricultural data (e.g. cereals production and yield)). The comparison showed that the CDI had performed better than the precipitation input alone. However, the relationship is complex due to the timing of drought stress and the specific agricultural effect.

Results of this analysis for several key regions in Jordan are shown in Table C2, which also evaluates the effect of a longer SPI-3 period (6 months). It shows the significant relationship in most instances and the generally better relationship between CDI and cereal yields than production.

Table C2 - Correlation between rainfed crop production and yield with CDI and SPI-3 for the period 1994-2016 [70].

| **Relationship^*^** | **Irbid** | **Ajloun** | **Jarash** | **Madaba** | **Karak** |
| --- | --- | --- | --- | --- | --- |
| **Production and yield vs CDI_6_^[[1]](#footnote-1)^** | | | | | |
| TP vs. CDI_6_ | ns** | ns | 0.48 | 0.21 | 0.54 |
| WP vs. CDI_6_ | 0.34 | ns | 0.40 | Ns | 0.50 |
| WY vs. CDI_6_ | 0.47 | 0.34 | 0.46 | Ns | 0.45 |
| BP vs. CDI_6_ | Ns | ns | 0.47 | 0.42 | 0.43 |
| BY vs. CDI_6_ | 0.26 | 0.26 | 0.30 | 0.18 | 0.65 |
| **Production and yield vs SPI-6** | | | | | |
| TP vs. SPI-6 | Ns | ns | 0.38 | 0.30 | 0.52 |
| WP vs. SPI-6 | 0.31 | ns | 0.30 | 0.21 | 0.47 |
| WY vs. SPI-6 | 0.34 | 0.34 | 0.32 | Ns | 0.41 |
| BP vs. SPI-6 | Ns | ns | 0.38 | 0.38 | 0.44 |
| BY vs. SPI-6 | 0.25 | 0.22 | 0.30 | 0.22 | 0.57 |
| **Production and yield vs CDI** | | | | | |
| TP vs. CDI^***^ | Ns | ns | 0.36 NJ | 0.22NJ*** | 0.47 DF |
| WP vs. CDI | 0.30 NJ | ns | 0.38 DF | Ns | 0.42 DF |
| WY vs. CDI | 0.47 NJ | 0.30 DF | 0.36 NJ | Ns | 0.38 JM |
| BP vs. CDI | Ns | ns | 0.24 NJ | 0.47NJ | 0.40 DF |
| BY vs. CDI | Ns | 0.23 DF | 0.23 NJ | 0.24NJ | 0.59 DF |
| **Production and yield vs SPI-3** | | | | | |
| TP vs. SPI-3^***^ | Ns | ns | 0.25 NJ | 0.27 NJ | 0.43 DF |
| WP vs. SPI-3 | 0.26 DF | 0.19 JM | 0.33 NJ | Ns | 0.40 NJ |
| WY vs. SPI-3 | 0.35 NJ | 0.25 JM | 0.20 NJ | Ns | 0.32 JM |
| BP vs. SPI-3 | Ns | ns | Ns | 0.42 NJ | 0.40 DF |
| BY vs. SPI-3 | Ns | ns | Ns | 0.23 NJ | 0.54 NJ |

* TP – total production; WP – wheat production; WY – wheat yield; BP – barley production; BY – barley yield

** ns – not significant at *P* < 0.05

*** Maximum significant seasonal correlation shown with the abbreviations NJ – Nov-Jan; DF – Dec-Feb; JM – Jan-Mar.

## Stage 2 CDI validation in Morocco

Table C3 shows the numerical percentage of Moroccan provinces for which the given month’s CDI class is the same as that of the annual wheat production anomaly. The production anomaly is calculated using the same percentile thresholds as the CDI per Section 2.4. The three years represent different types of drought years.

Table C3 - Match between CDI and wheat production anomaly for Moroccan provinces

| Month | 2000-2001 (middle of a multi-year drought) | 2004-2005 (single drought year) | 2006-2007 (first year of a two-year drought) |
| --- | --- | --- | --- |
| Oct | 17.9% | 53.0% | 33.1% |
| Nov | 15.4% | 49.8% | 25.2% |
| Dec | 28.2% | 41.9% | 13.4% |
| Jan | 35.9% | 29.2% | 7.6% |
| Feb | 17.9% | 24.4% | 31.3% |
| Mar | 7.6% | 51.3% | 26.4% |
| Apr | 7.6% | 40.3% | 42.6% |
| May | 2.5% | 34.0% | 23.1% |

Subsequent analysis in Morocco not reported in this paper highlighted that different agro-ecological zones tended to have different periods of alignment between the CDI and production anomalies, and [17] evaluated the Stage 0 CDI in relation to cereal yields in Morocco including detailed case studies of two regions.

## Stage 2 semi-quantitative evaluation in Tunisia

Early Stage 2 CDI validation in Tunisia (December 2017 to April 2018) included surveying a local agriculture official in each governorate about their perceptions of the accuracy of the monthly CDI maps in relation to geographic area and drought intensity. Response rates and locations varied significantly from month to month, so the data couldn’t be examined in a structured fashion. However, there was consistent feedback about the CDI “noise” as described in Section 3.2.2 that resulted in stakeholders’ decreased perceptions of CDI accuracy. Also, numerous comments about the intra-governorate distribution of drought impacts, both geographically and by sector, were useful for considering future improvements to the CDI and its interpretation by policy decision-makers.

# Supplementary Information D – Additional results related to precipitation including comparison of CHIRPS and IMERG to observation station data in Jordan, Lebanon, and Tunisia

## Stage 1 precipitation analysis (Jordan and Morocco)

Note that this analysis was undertaken in Stage 1 and preceded the analysis shown in Table 2 and remainder of this Supplementary Information Section, which was undertaken in Stage 2.

In Jordan and Morocco, CHIRPS precipitation data compared favorably to station data, particularly in the rainy season, which coincides with the agricultural year.

Table D1 below shows the coefficient of determination between the annual and rainy season CHIRPS-derived and station SPI-3 values in Jordan. Overall, better performance was achieved during the rainy season, and more particularly in highlands areas that have higher annual precipitation and are the centre of rainfed agricultural production.

Table D1 - Correlation between CHIRPS-derived SPI-3 and Jordanian observation station SPI-3 values

| **Station** | **Annual rainfall (mm)** | **R^2^ for all year SPI-3** | **R^2^ for Rainy season** |
| --- | --- | --- | --- |
| Irbid | 460 | 0.50 | 0.68 |
| ErRabeh | 337 | 0.28 | 0.52 |
| Amman Airport | 254 | 0.34 | 0.59 |
| Tafileh | 204 | 0.29 | 0.43 |
| Queen AIA | 156 | 0.29 | 0.46 |
| Zarqa | 129 | 0.28 | 0.46 |
| Ruwayshid -H4 | 81 | 0.52 | 0.57 |
| Safawi-H5 | 70 | 0.27 | 0.39 |
| Azraq | 54 | 0.13 | 0.21 |
| Al Jafer | 31 | 0.21 | 0.29 |

In Morocco, Maroc Meteo and the International Center for Biosaline Agriculture [72] evaluated 1981-2017 precipitation data from 30 ground stations in relation to CHIRPS and found a high correlation during the agricultural year at almost all stations, as shown in Table D2 below.

Table D2 – Mean Error (ME), Root Mean Square Error (RMSE) and correlation coefficient (r) between observed precipitation at noted rain stations and CHRIPS data for the months of October-November, December-January, and February March covering the period from 1981 to 2017

## Stage 2 precipitation analysis in Lebanon

*Comments on data quality:*

The available data from Lebanese stations has a limited period, ranging from 7 to 16 years, with many gaps for most stations. The quality of the data is medium to poor, with the exception of Beirut airport, which has minimal gaps and good continuity.

**Table D3 –** Assessment of CHIRPS and IMERG precipitation data in relation to select Lebanese observation stations.

| **Station** | **Period** | **Correlation (R)** | | **RMSE** | |
| --- | --- | --- | --- | --- | --- |
|  |  | **CHIRPS** | **IMERG** | **CHIRPS** | **IMERG** |
| Abdeh | 2001-2015 | 0.91 | 0.80 | 100.63 | 104.68 |
| Beirut airport | 2001-2018 | 0.94 | 0.75 | 26.90 | 65.02 |
| Daher elbaider | 2004-2014 | 0.64 | 0.61 | 68.08 | 64.97 |
| Deir Al Ahmar | 2001-2014 | 0.87 | 0.46 | 34.25 | 164.02 |
| Deir El kamar | 2005-2016 | 0.92 | 0.68 | 44.44 | 82.32 |
| Faqra | 2006-2015 | 0.27 | 0.21 | 109.12 | 128.64 |
| Hermel | 2005-2016 | 0.72 | 0.53 | 55.85 | 114.07 |
| Jezzine | 2006-2015 | 0.88 | 0.64 | 64.98 | 104.59 |
| Kfarchakhna | 2001-2014 | 0.87 | 0.69 | 41.90 | 68.26 |
| Kfardounine | 2009-2016 | 0.83 | 0.74 | 52.53 | 66.09 |
| Kfarqouq | 2006-2014 | 0.84 | 0.73 | 43.05 | 60.57 |
| Lebaa | 2005-2014 | 0.81 | 0.65 | 56.92 | 75.49 |
| Marjayoun | 2010-2016 | 0.81 | 0.69 | 57.37 | 75.03 |
| Qartba | 2001-2015 | 0.74 | 0.66 | 75.87 | 90.74 |
| Qousaiyba | 2005-2012 | 0.84 | 0.67 | 51.11 | 75.75 |
| Qoubayat | 2001-2016 | 0.85 | 0.62 | 55.95 | 78.28 |
| Quaoroun | 2003-2015 | 0.78 | 0.65 | 60.96 | 74.56 |
| Riak | 2001-2016 | 0.60 | 0.27 | 64.42 | 69.79 |
| Saida | 2001-2013 | 0.85 | 0.78 | 50.58 | 49.06 |
| Seir eldenneiah | 2009-2014 | 0.84 | 0.62 | 52.75 | 104.28 |
| Sour | 2007-2018 | 0.83 | 0.78 | 51.46 | 52.24 |
| Tripoli | 2001-2018 | 0.93 | 0.76 | 31.90 | 64.18 |
| Zahleh | 2001-2015 | 0.96 | 0.72 | 23.16 | 74.43 |

CHIRPS data has a better correlation to observed in situ rainfall data compared to IMERG. CHIRPS R scores show a strong correlation in most stations, reaching over 90% for some, such as Beirut airport, Deir Alkamar and Zahleh. Additionally, the CHIRPS data has smaller root mean square errors, which further reinforces the level of confidence in its accuracy for Lebanon.

## Stage 2 precipitation analysis in Jordan

*Comments on data quality:*

The Jordanian dataset spans 21 years, with the exception of one station that started operation in 2005. The data generally has good continuity, with some gaps and missing data for certain stations. The stations have excellent representativity for the entire territory and cover all of the country's diverse climate zones.

**Table D4 –** Assessment of CHIRPS and IMERG precipitation data in relation to select Jordanian observation stations.

| **Stations** | **Period** | **Correlation** | | **RMSE** | |
| --- | --- | --- | --- | --- | --- |
|  |  | **CHIRPS** | **IMERG** | **CHIRPS** | **IMERG** |
| Baqura | Jan 2001 – Apr 2021 | 0.90 | 0.79 | 26.80 | 32.39 |
| Deir Alla | Jan 2001 – Apr 2021 | 0.90 | 0.74 | 20.21 | 23.37 |
| Ghor Safi | Jan 2001 – Dec 2018 | 0.59 | 0.60 | 8.49 | 18.32 |
| Irbed | Jan 2001 – Apr 2021 | 0.90 | 0.67 | 26.49 | 43.61 |
| Er Rabbah | Jan 2001 – Apr 2021 | 0.82 | 0.48 | 24.86 | 38.78 |
| Shoubak | Jan 2001 – Apr 2021 | 0.71 | 0.42 | 20.09 | 27.32 |
| Wadi Dhulall | Jan 2001 – Apr 2021 | 0.86 | 0.56 | 8.94 | 14.24 |
| Samma | Jan 2005 – Apr 2021 | 0.91 | 0.74 | 29.51 | 42.20 |
| Wadi El-rayyan | Jan 2001 – Apr 2021 | 0.89 | 0.72 | 19.81 | 37.65 |
| Swaileh | Jan 2001 – Apr 2021 | 0.90 | 0.60 | 42.07 | 52.08 |
| Qatraneh | Jan 2001 – Apr 2021 | 0.74 | 0.48 | 11.96 | 21.73 |
| Tafileh | Jan 2001 – Apr 2021 | 0.51 | 0.18 | 23.72 | 30.83 |
| Zarqa | Jan 2001 – Apr 2021 | 0.90 | 0.65 | 7.73 | 19.83 |
| Salt | Jan 2001 – Apr 2021 | 0.90 | 0.65 | 42.91 | 58.24 |
| KH Airport | Jan 2001 – Apr 2021 | 0.52 | 0.39 | 4.41 | 16.43 |
| Ras Muneef | Jan 2001 – Apr 2021 | 0.93 | 0.68 | 36.46 | 56.94 |
| Amman Airport | Jan 2001 – Apr 2021 | 0.92 | 0.66 | 15.43 | 24.58 |
| Rwaished (H4) | Jan 2001 – Apr 2021 | 0.79 | 0.70 | 6.98 | 16.83 |
| Mafraq | Jan 2001 – Apr 2021 | 0.88 | 0.65 | 8.38 | 13.64 |
| Safawi (H5) | Jan 2001 – Apr 2021 | 0.71 | 0.68 | 6.40 | 11.73 |
| Azraq South | Jan 2001 – Apr 2021 | 0.62 | 0.54 | 7.15 | 11.88 |
| Q.A.I.Airport | Jan 2001 – Apr 2021 | 0.87 | 0.57 | 11.86 | 19.22 |
| Ma'an | Jan 2001 – Apr 2021 | 0.64 | 0.51 | 4.55 | 12.25 |
| Al Jafer | Jan 2001 – Apr 2021 | 0.40 | 0.53 | 6.34 | 11.06 |
| Ghabawi | Jan 2005 - Apr 2021 | 0.82 | 0.59 | 8.66 | 12.83 |

The CHIRPS data has better performance and correlation with observation data in Jordan compared to IMERG. CHIRPS correlation reaches up to 90% for some stations. IMERG's correlation is still acceptable but lower, mainly ranging from 50-80%. Additionally, the root mean square error values for CHIRPS are smaller than those for IMERG. Together, the higher correlation values and smaller error values confirm that CHIRPS data is a more reliable representation of rainfall in Jordan compared to IMERG's data estimation.

## Stage 2 precipitation analysis in Tunisia

*Comments on data quality:*

The Tunisian dataset has the highest quality of the three countries. Many stations have no missing data at all, with excellent continuity, and many have only a small number of missing values or gaps. Although there are fewer stations used in comparison to Jordan and Lebanon, they still represent all the climate zones of the country, from the desert in the south to the humid and subhumid regions in the northwest.

**Table D5 –** Assessment of CHIRPS and IMERG precipitation data in relation to select Tunisian observation stations.

| **Stations** | **Period** | **Correlation** | | **RMSE** | |
| --- | --- | --- | --- | --- | --- |
|  |  | CHIRPS | IMERG | CHIRPS | IMERG |
| **Tabarka** | Jun 2000 – Dec 2020 | 0.91 | 0.54 | 36.14 | 99.79 |
| **Bizerte** | Jun 2000 – Dec 2020 | 0.89 | 0.51 | 25.89 | 113.26 |
| **Tunis** | Jun 2000 – Dec 2020 | 0.87 | 0.65 | 23.87 | 114.69 |
| **Zaghouan** | Jun 2000 – Dec 2020 | 0.80 | 0.41 | 26.15 | 98.27 |
| **Kelibia** | Jun 2000 – Dec 2020 | 0.87 | 0.60 | 22.76 | 122.79 |
| **Jendouba** | Jun 2000 – Dec 2020 | 0.89 | 0.42 | 18.52 | 66.10 |
| **Beja** | Jun 2000 – Dec 2020 | 0.89 | 0.49 | 22.31 | 72.51 |
| **Kasserine** | Jun 2000 – Dec 2020 | 0.76 | 0.48 | 12.93 | 55.71 |
| **Kairouan** | Jun 2000 – Dec 2020 | 0.83 | 0.57 | 15.58 | 67.79 |
| **Sidi-bouzid** | Jun 2000 – Dec 2020 | 0.79 | 0.57 | 13.85 | 46.51 |
| **Monastir** | Jun 2000 – Dec 2020 | 0.86 | 0.66 | 19.06 | 94.65 |
| **Sfax** | Jun 2000 – Dec 2020 | 0.80 | 0.66 | 14.22 | 51.35 |
| **Gabes** | Jun 2000 – Dec 2020 | 0.73 | 0.36 | 16.61 | 64.83 |
| **Gafsa** | Jun 2000 – Dec 2020 | 0.79 | 0.55 | 10.43 | 26.88 |
| **Medenine** | Jun 2000 – Dec 2020 | 0.72 | 0.62 | 17.50 | 33.32 |

The results for Tunisia are similar to those for Jordan and Lebanon. CHIRPS data shows clearly better performance in estimating monthly rainfall across all Tunisian stations, despite the wide range of climate characteristics. CHIRPS correlation values are high for all stations, reaching 90% for some stations in the north. None of the values are below 70. On the other hand, IMERG data has poor performance and low correlation values for most stations, with correlations below 50% for many stations. Additionally, comparing the root mean square error values for both satellite sources, CHIRPS has better performance, with small error values compared to IMERG.

1. The “CDI-6” used SPI-6 rather than SPI-3 as the precipitation input index. [↑](#footnote-ref-1)
