# Supplementary material for: Development of a composite drought indicator for operational drought monitoring in the MENA region
Source: Sci Rep. 2024 Mar 5;14:5414. doi: 10.1038/s41598-024-55626-0 (PMC10914844; doi:10.1038/s41598-024-55626-0)
Supplement: Supplementary file 2 — Supplementary Information 2. [file 41598_2024_55626_MOESM2_ESM.pdf]

Drought Analysis Application for Jordan

User Guide

App dashboard

Analysis by administrative region

Analysis by watershed

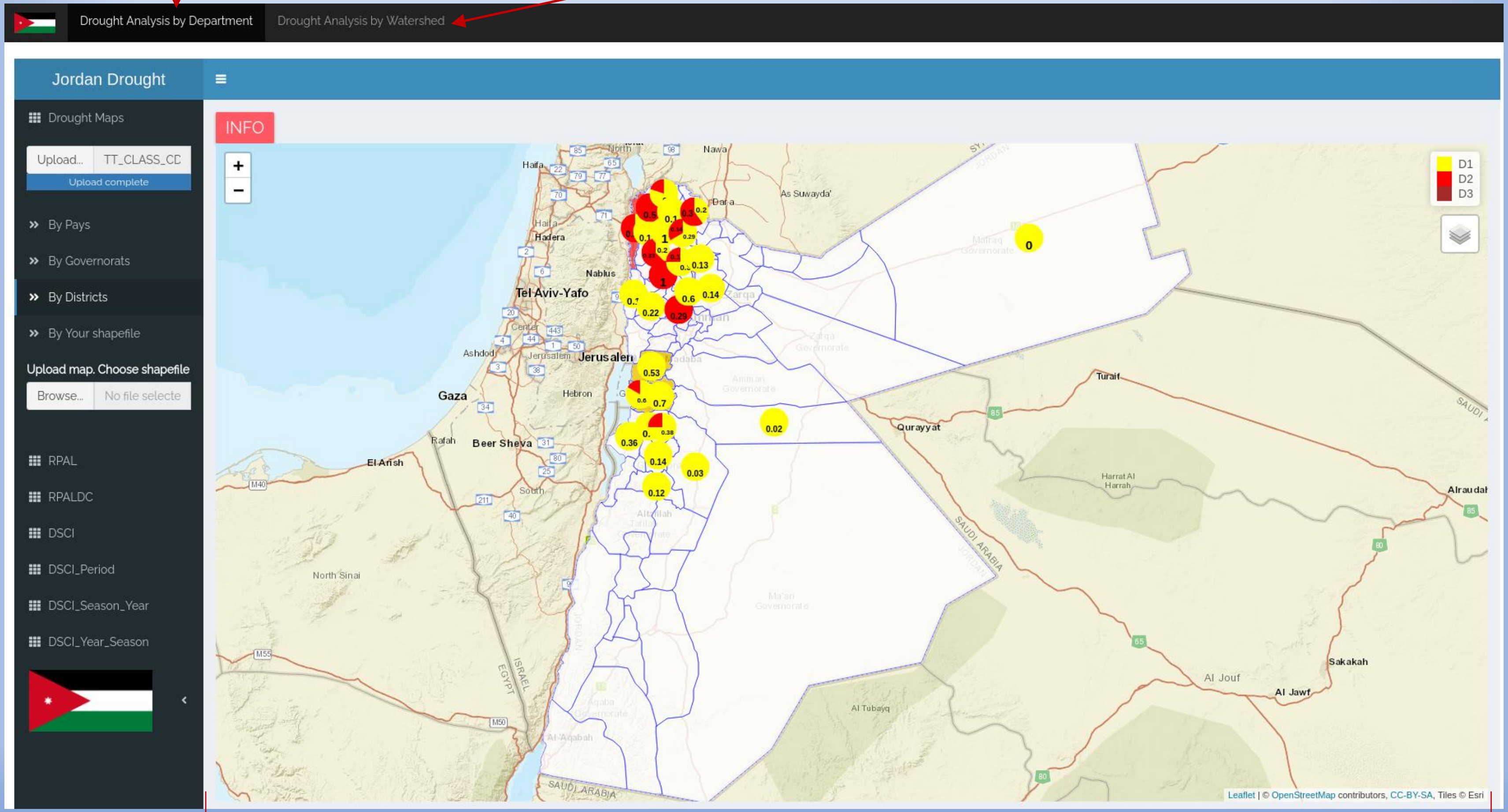

Control Panel

Output

Control Panel

First Step: You must choose the monthly drought file (.nc)

The first tab presents the drought map for all the country
The second tab presents the drought map by Governorate
The third tab presents the drought map by District

By your shapefile: Tools to create and export shapefiles and draw the drought map over the specific shp

Upload map. Choose shapefile: Tools to upload shapefiles created to Calculate and visualise the drought map only over this shp file.

RPAL: ***Regional Percent Drought Area***

RPALDC: ***Regional Percent Area Drought Cumululative***

DSCI: ***Drought Severity And Coverage Index***

DSCI_Period: ***Drought Severity And Coverage Index By Period***

DSCI_Season_Year: ***Boxplot of DSCI by coupled variable (Year-Season)***

DSCI_Year_Season: ***Boxplot of DSCI by coupled variable (Year-Season) grouped by year***

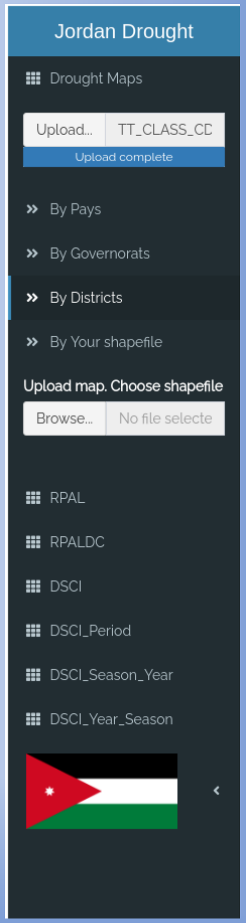

First Step: You must choice netcdf file

Once you click on Upload, a new window will appear to select the netcdf file relevant for the analysis.

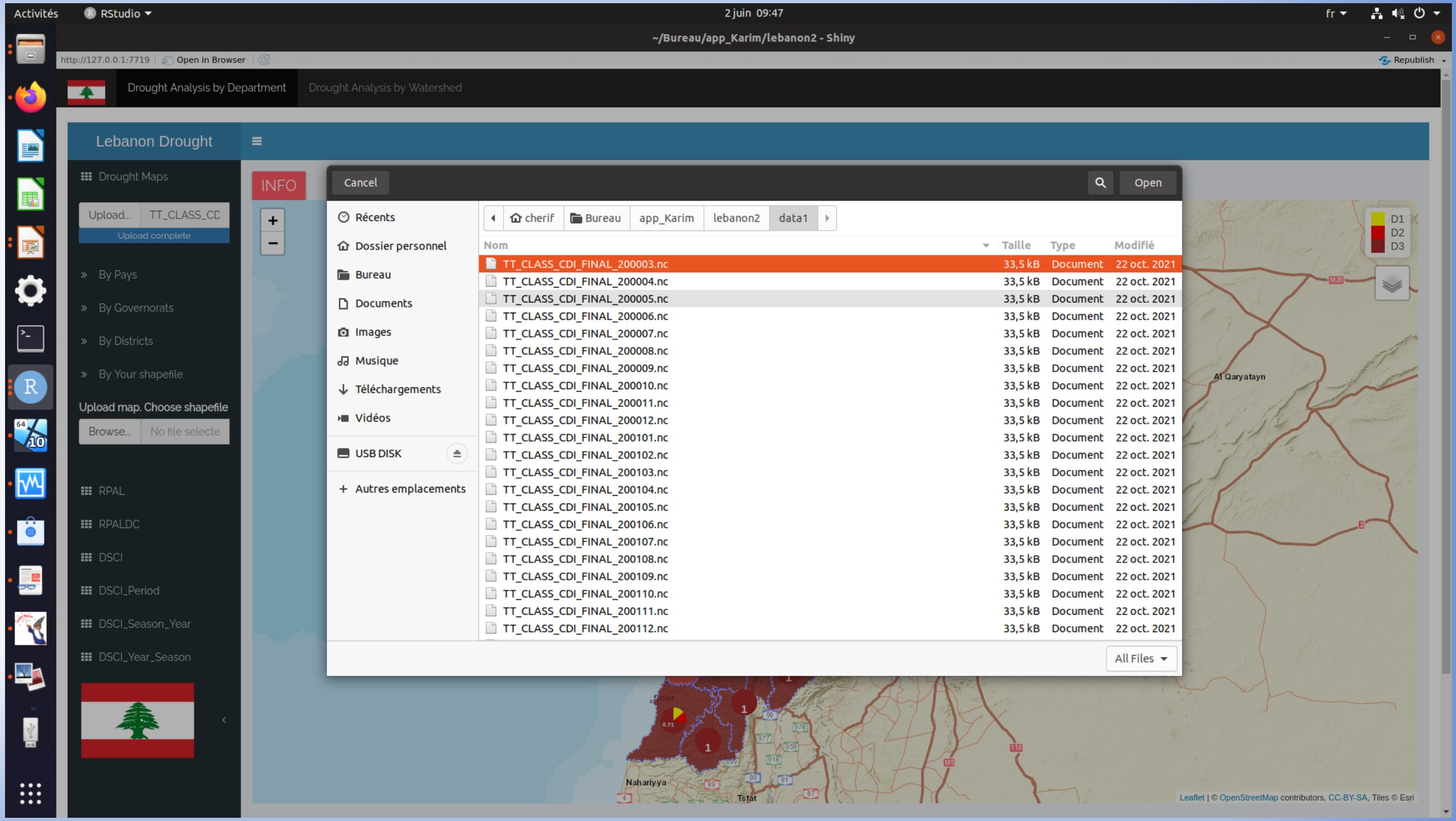

Drought map for Jordan Country

The first output is the drought map from the data of the whole country calculated with the indicators D1, D2, D3.

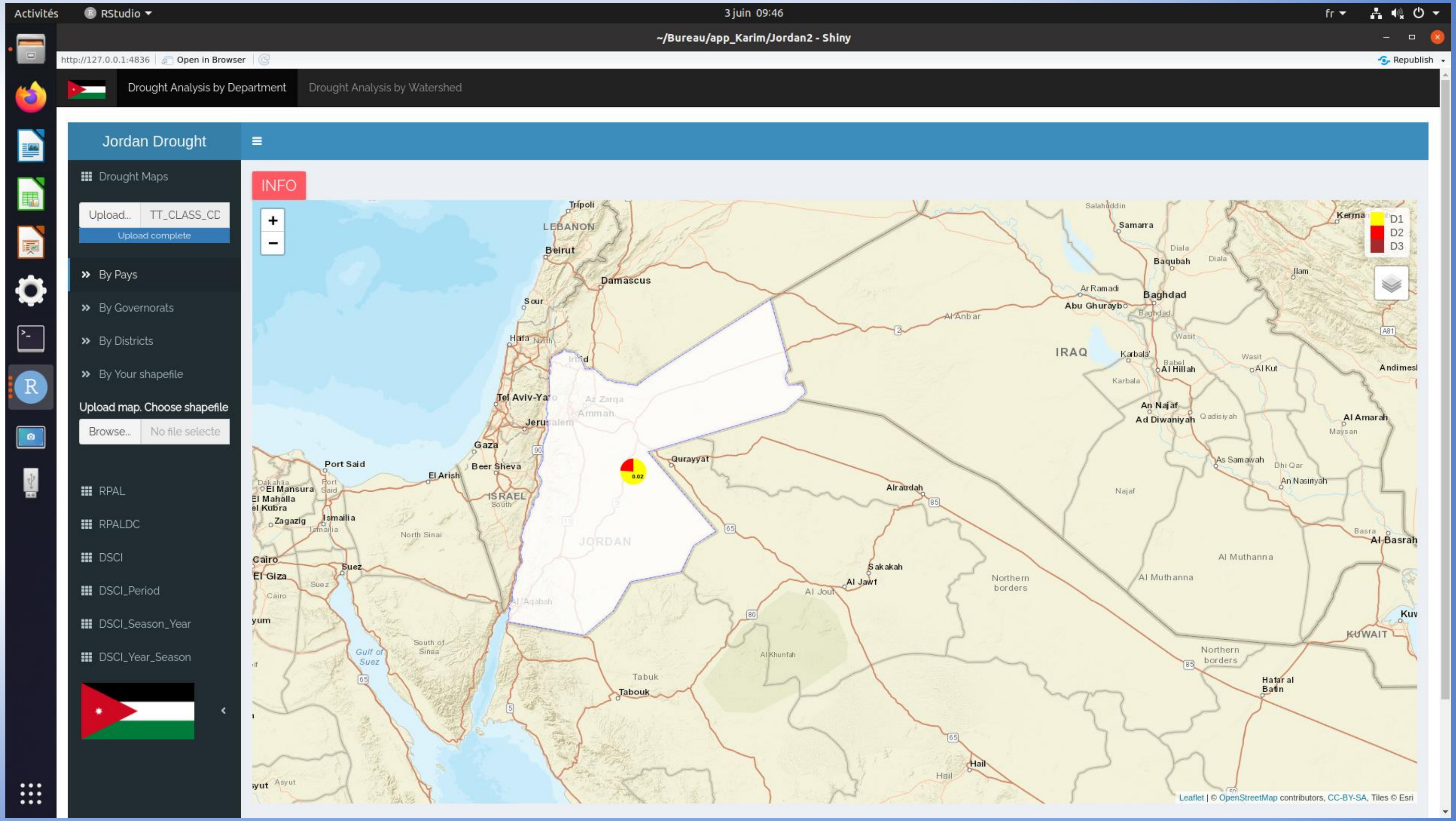

Drought map by Governorates

At this level, the drought map is redrawn by governorates, and the drought indicators are calculated for each governorate.

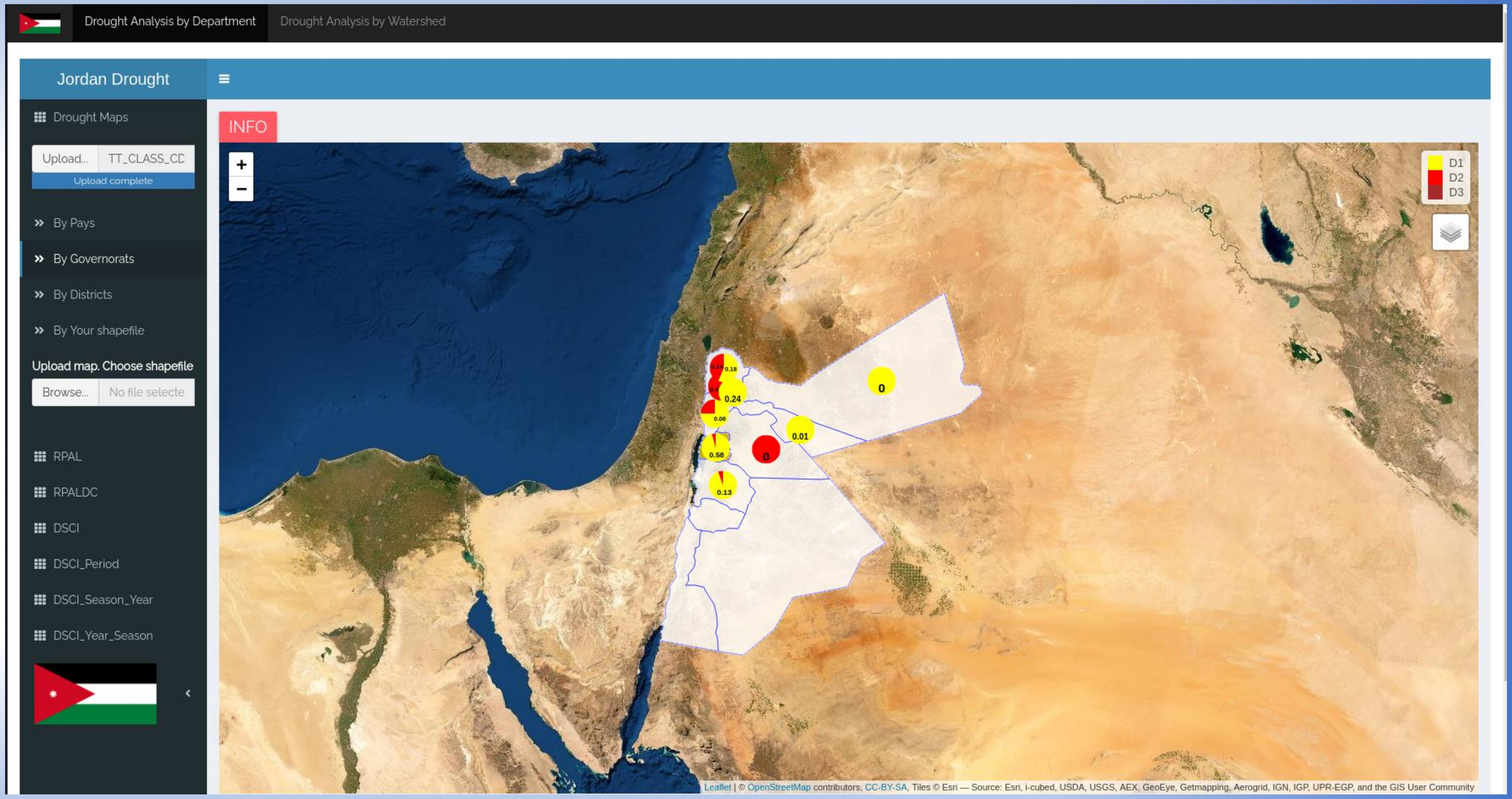

Drought Map by Districts

At this level, the drought map is redrawn by districts, and the drought indicators are calculated for each district.

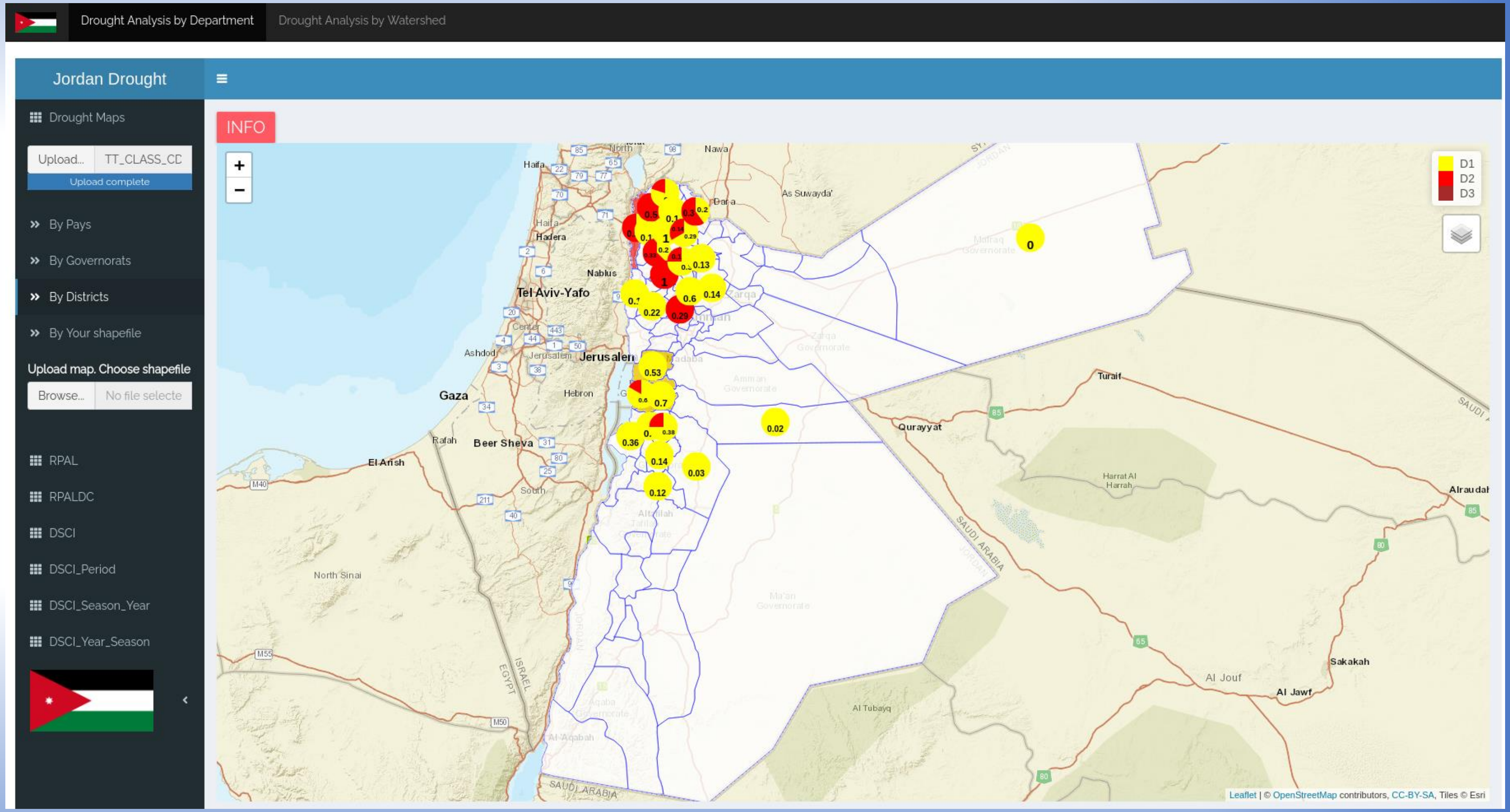

Tools to draw, create and export shp file

Select this icon to draw a new polygon

New Polygon created

Button to export polygon as shp file

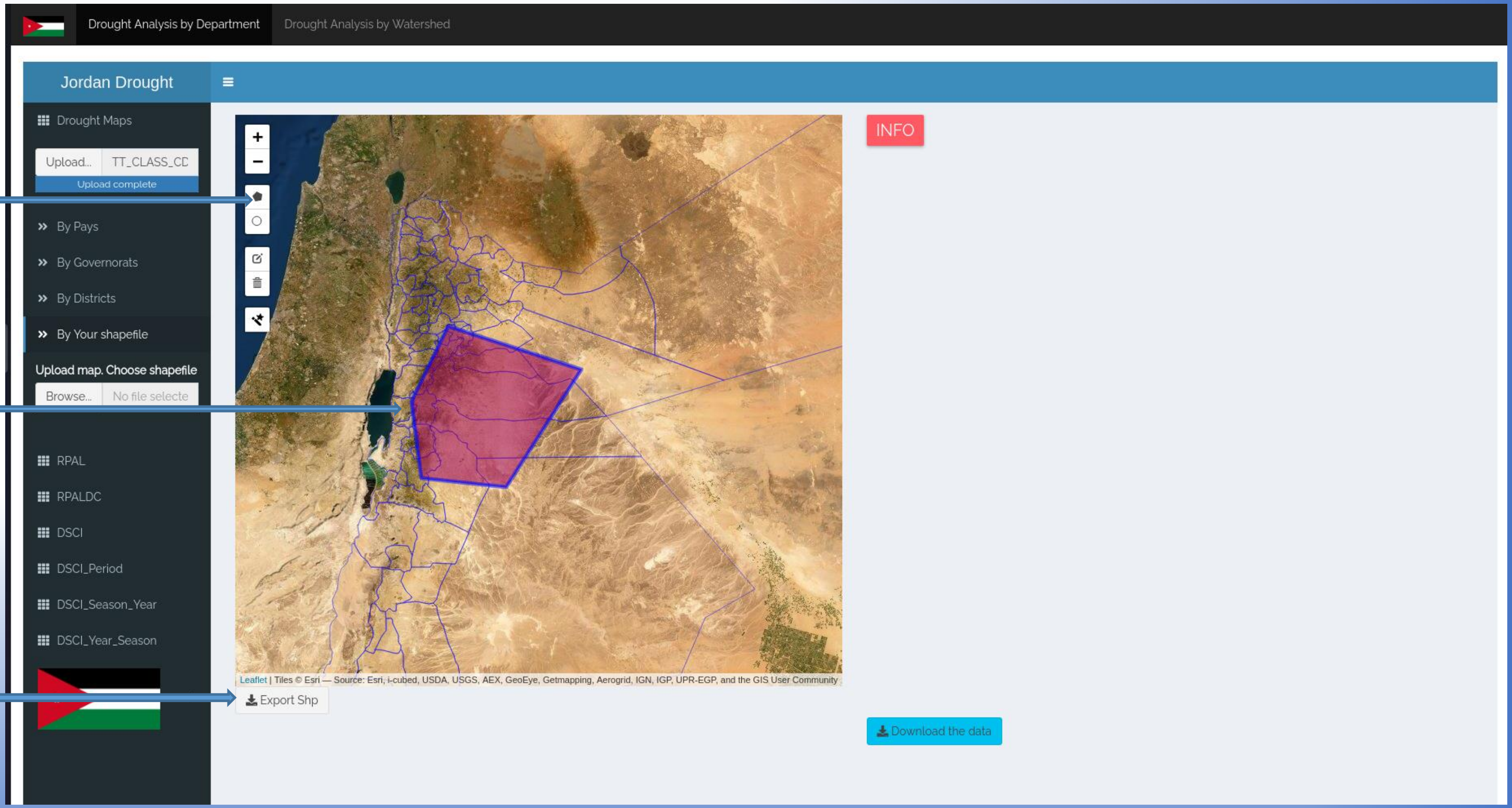

Tools to import shp file and draw drought map

Extract shpExport.zip file to obtain 4 files (.shp, .prj, .dbf, .shx)

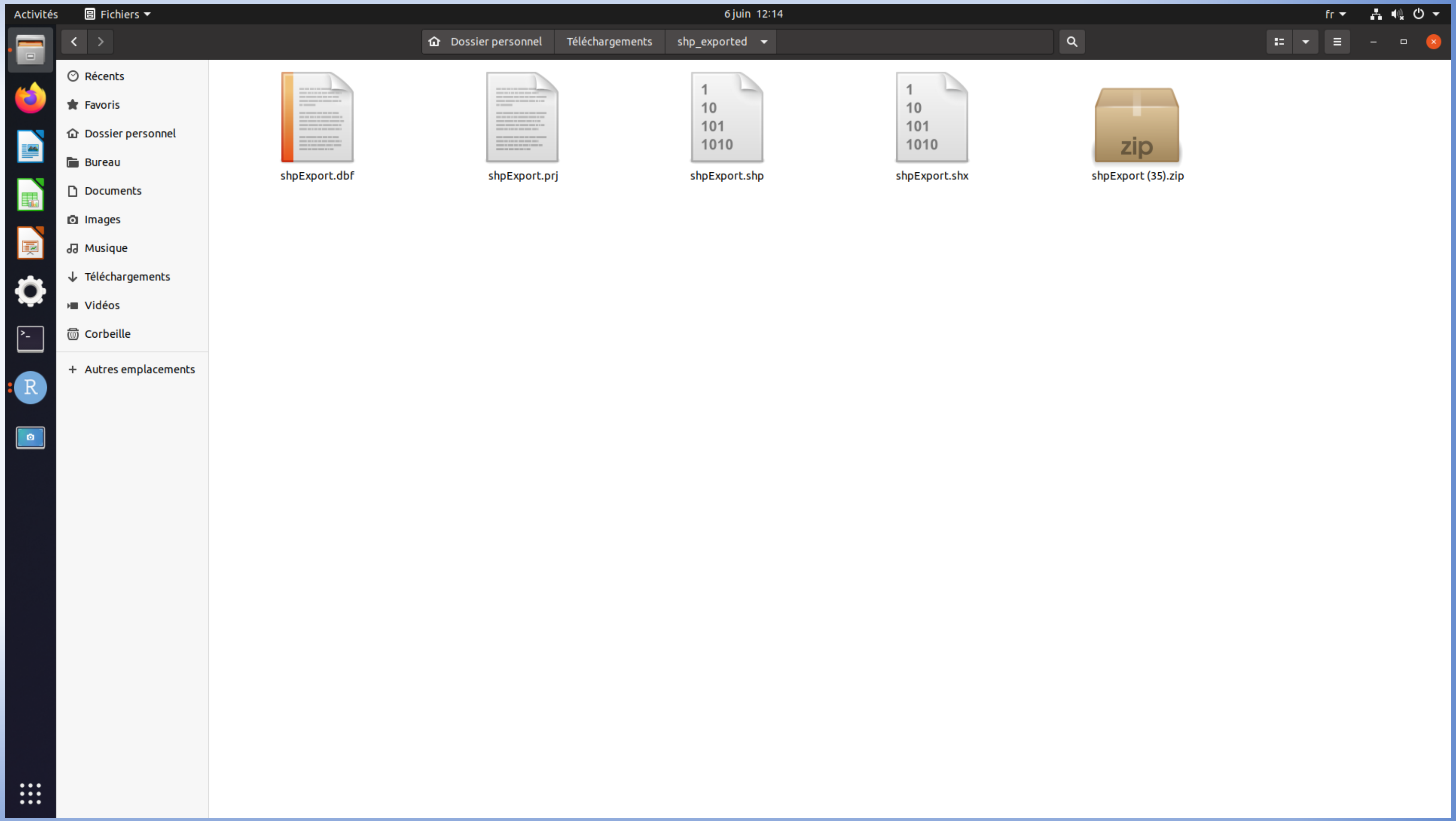

Tools to import shp file and draw drought map

Click here to open a new window,
Select 4 files and clic open

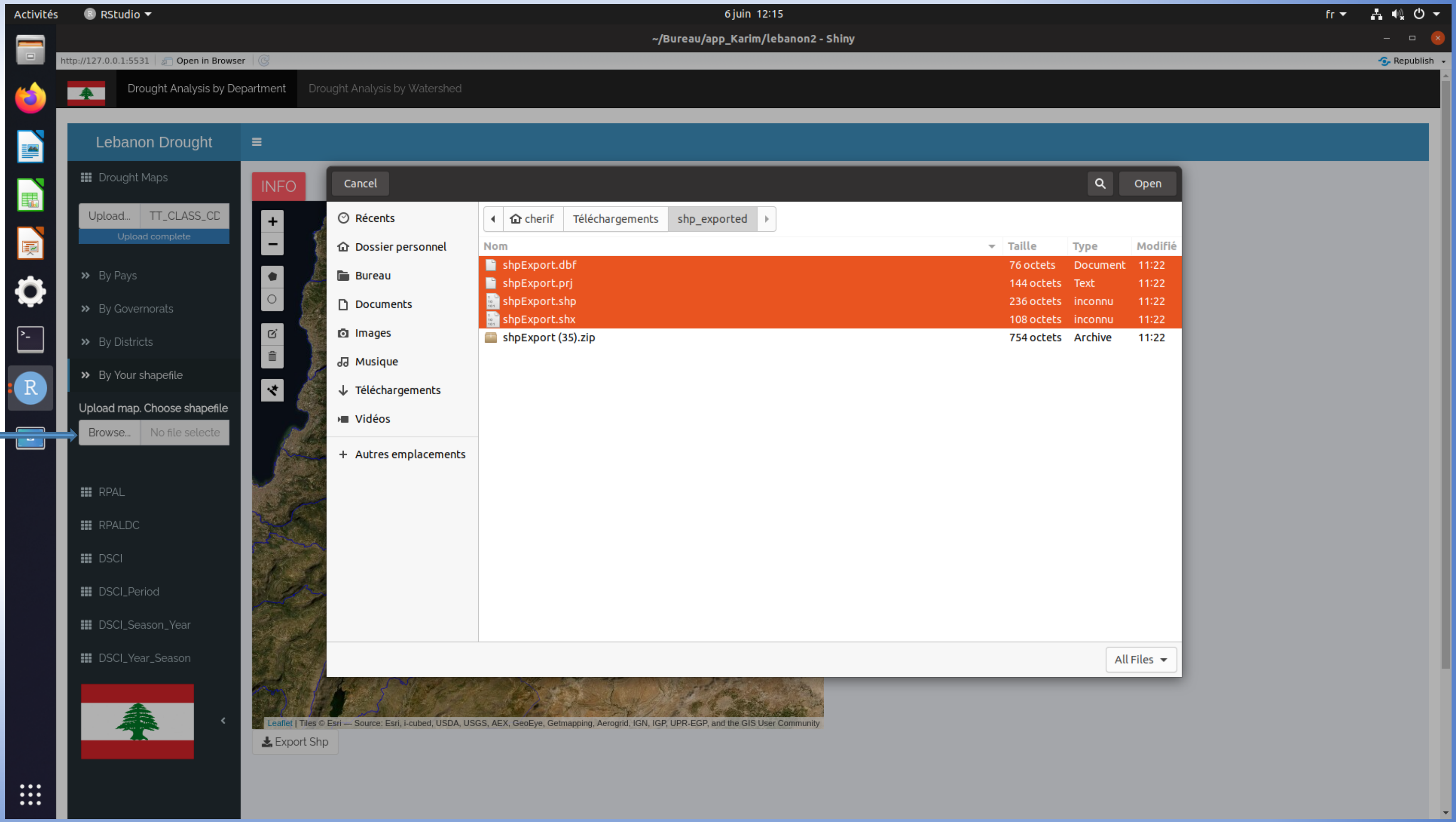

Tools to import shp file and draw drought map

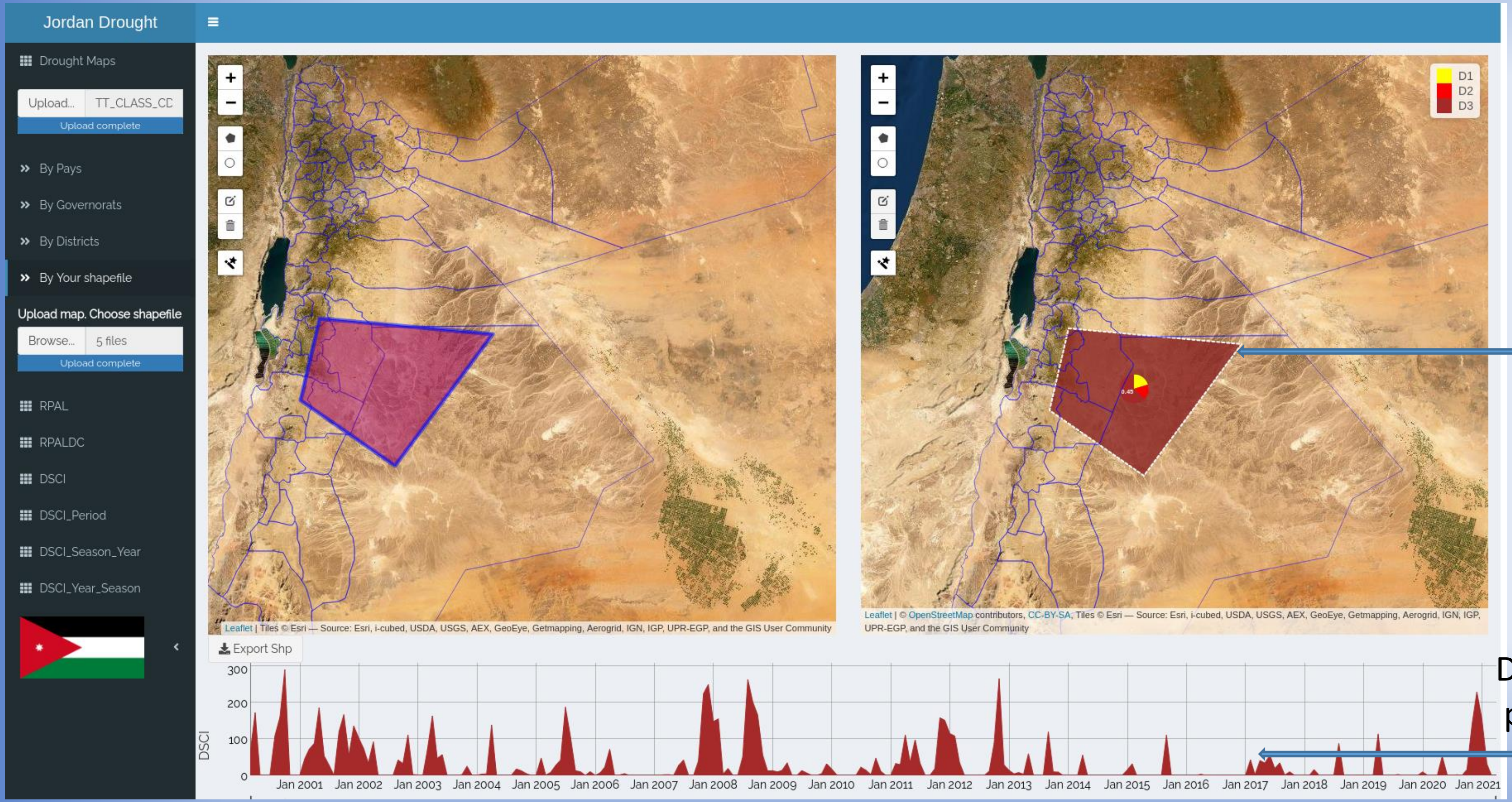

Drought map for the specific shp file imported

DSCI calculated from the imported polygon over the entire analysis period.

Drought Indicators

- The drought classes are between -3 and +3

✓ -3 : Exceptional Drought

✓ -2 : Severe Drought

✓ -1 : Moderate Drought

✓ 0 : Normal

✓ 1 : Moderate Wet

✓ 2 : Severe Wet

✓ 3 : Exceptional Wet

Drought Indicators

- D3: Percent area of Exceptional Drought class
- D2: Percent area of Severe Drought class
- D1: Percent area of Moderate Drough class
- D0: Percent area of Normal class
- D11: Percent area of Moderate Wet class
- D22:Percent area of Severe Wet class
- D33: Percent area of Exceptional Wet class

Regional Percent Drought Area

Select Area

Download csv data

Time Series for D1, D2 and D3

Same tools used to compare two regions

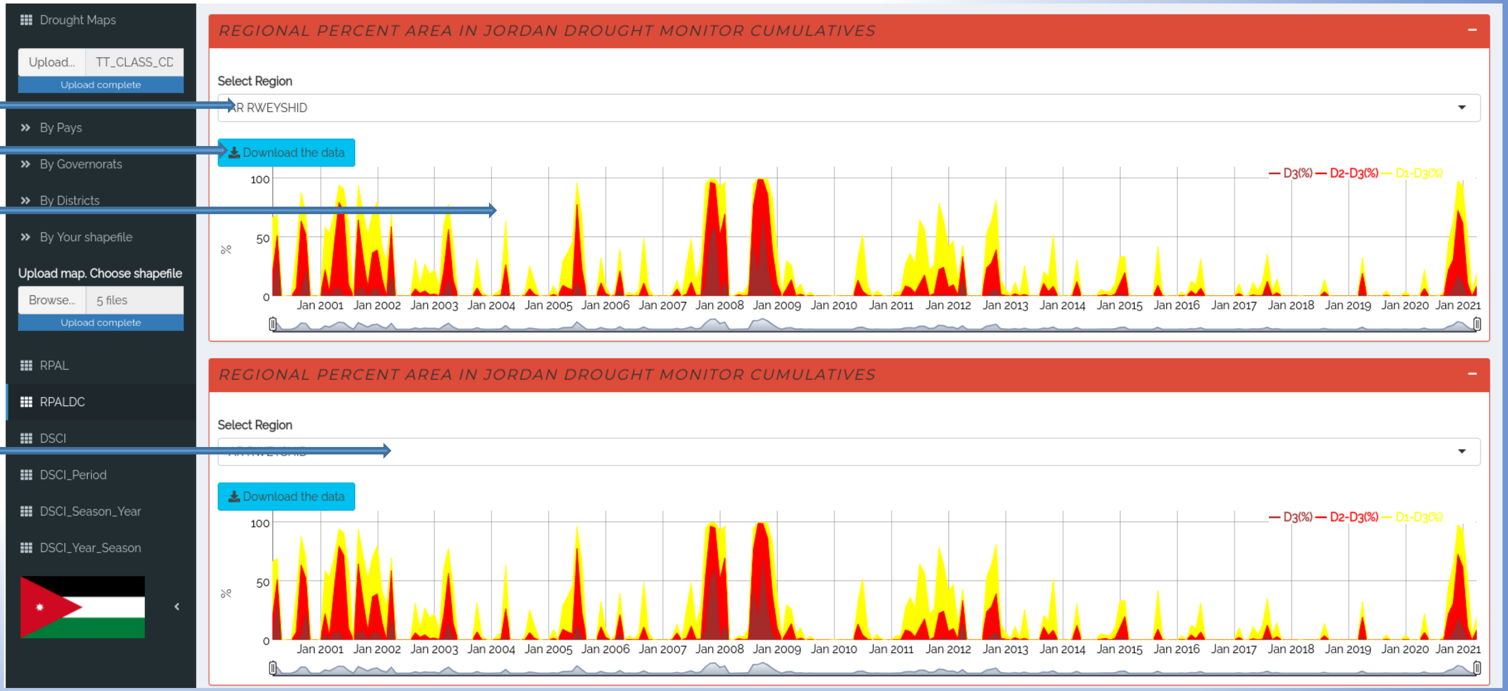

Regional Percent Area Drought Cumululatives

Intervals between indicators are used as a class to calculate the percentage of cumulative drought area

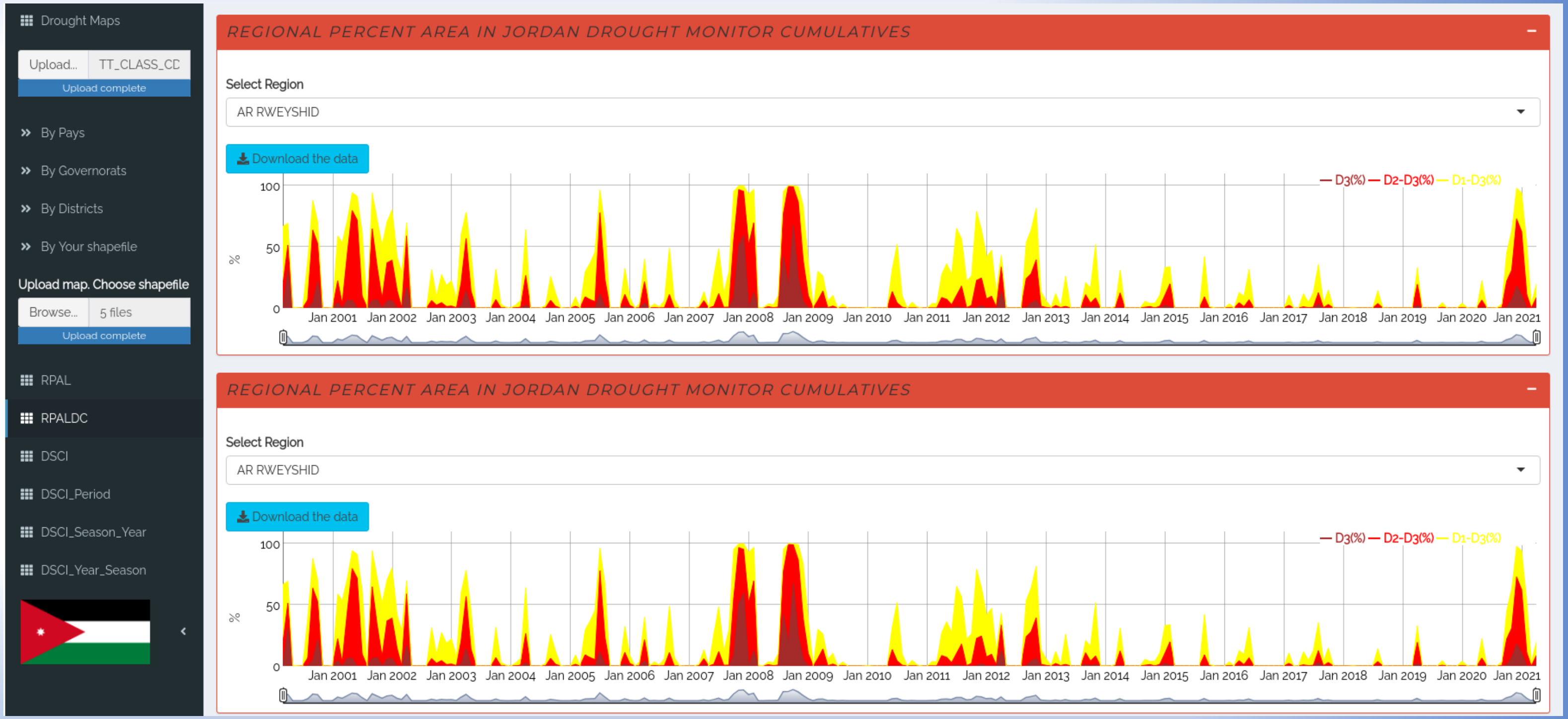

Drought Severity And Coverage Index DSCI

$$DSCI = \sum_{k=1}^{3} kD_k$$

Where: $0 \leq DSCI \leq 300$

$0 \leq D_k \leq 100$

Drought Severity And Coverage Index DSCI

Select Area

Download csv data

DSCI

Same tools used to compare two regions

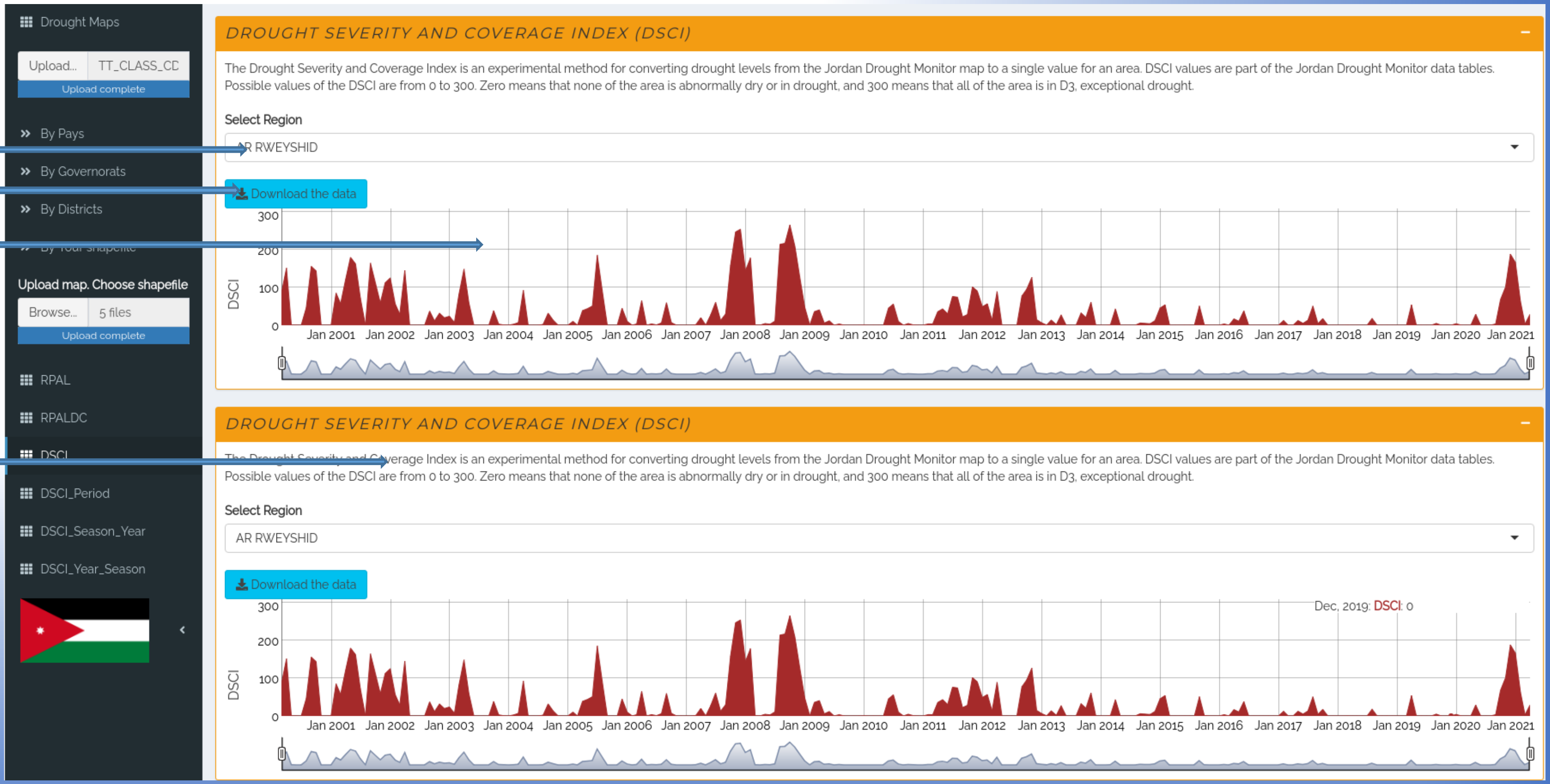

Drought Severity And Coverage Index DSCI By Period

Setting to select period and region to calculate their DSCIs

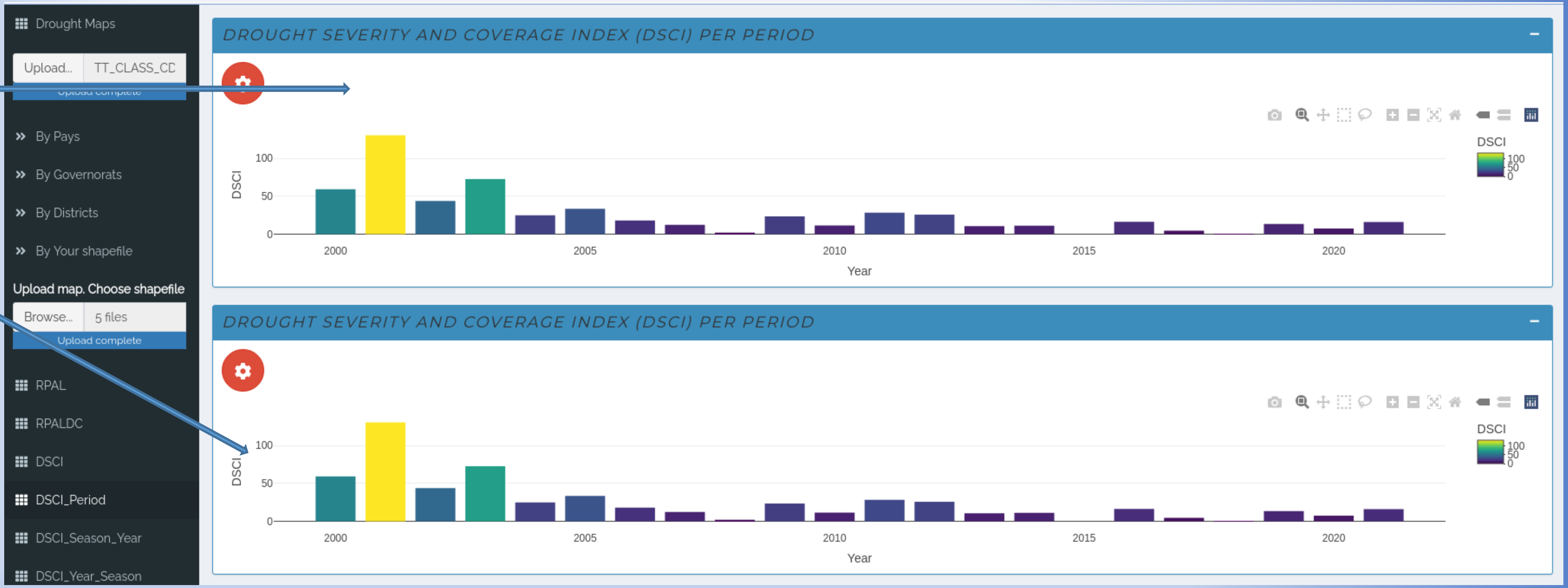

Boxplot of DSCI by coupled variable (Year-Season)

Setting allows you to filter data by season and year for each region selected and draw their boxplots

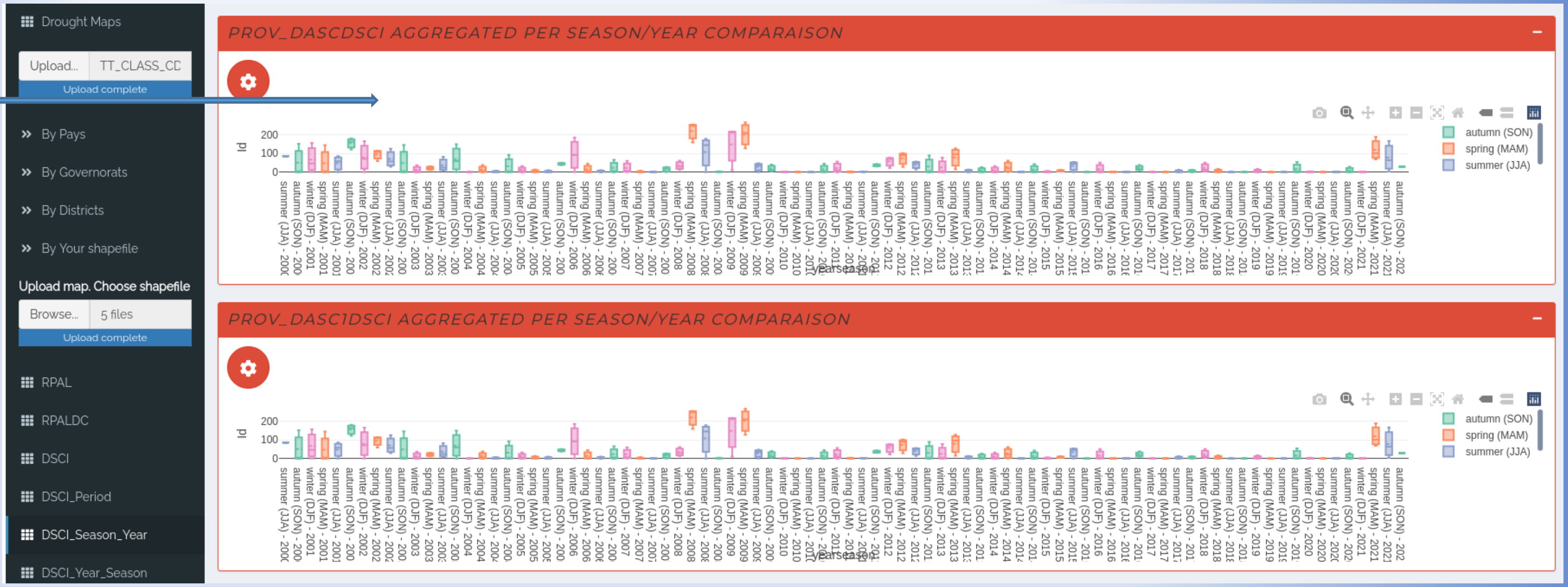

Boxplot of DSCI by coupled variable (Year-Season) grouped by year

Setting allows you to filter data by season of each region selected and draw their boxplots grouped by year

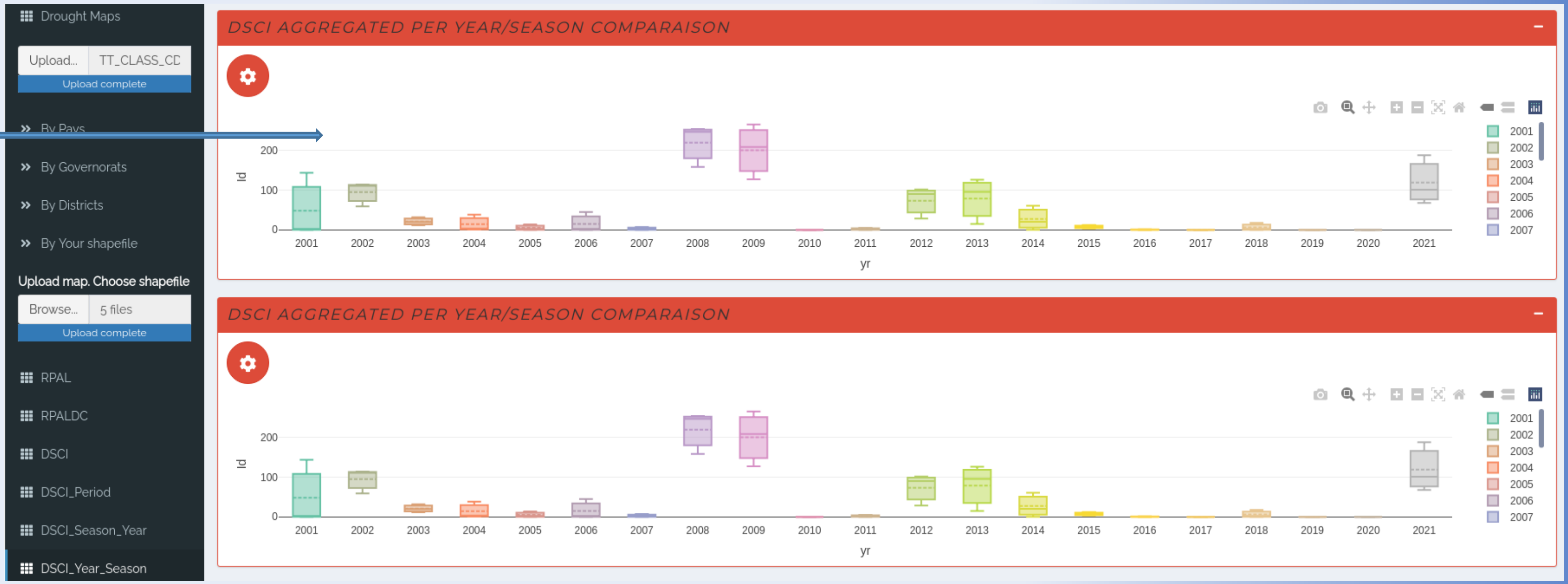

Drought Analysis By Watershed

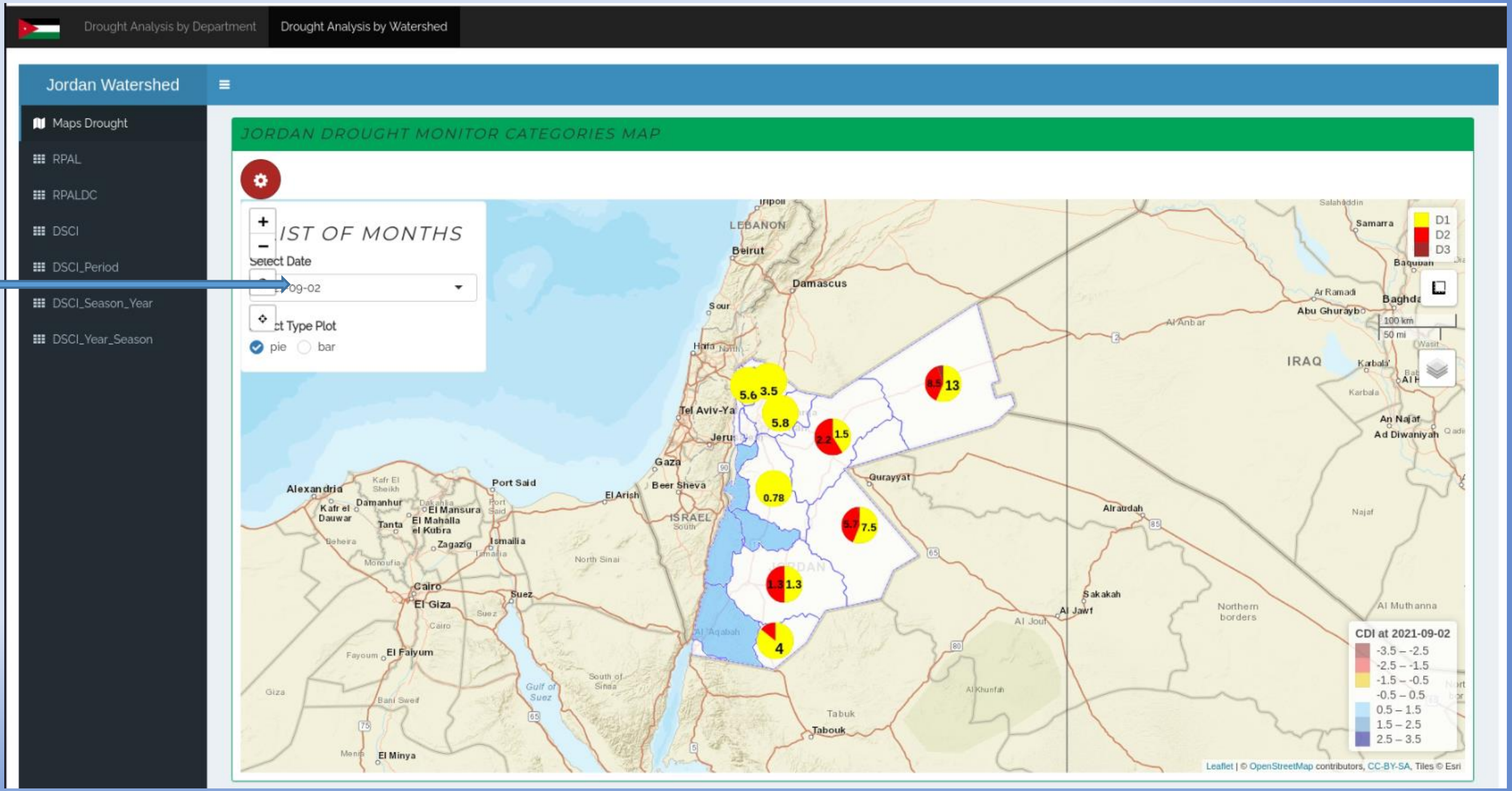

Select date to plot the corresponding drought map

Others Tools

- *All others tools developed for administrative regions are also developed for watershed areas.*

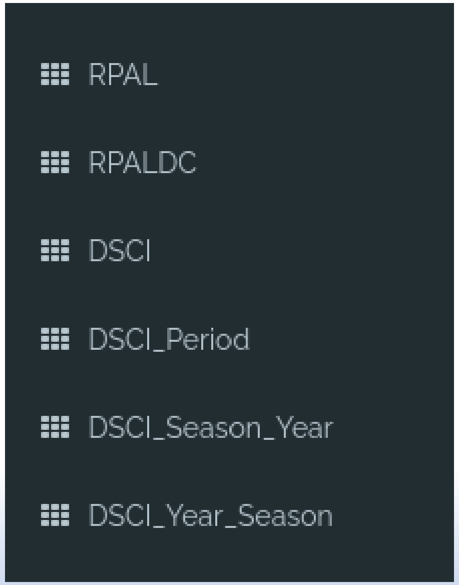

Thank you
